# Gut microbiota and ankylosing spondylitis: current insights and future challenges

**DOI:** 10.15698/mic2025.08.857

**Published:** 2025-08-25

**Authors:** Andrei Lobiuc, Liliana Groppa, Lia Chislari, Eugeniu Russu, Marinela Homitchi, Camelia Ciorescu, Sevag Hamamah, I. Codruta Bran, Mihai Covasa

**Affiliations:** 1 Department of Biological and Morphofunctional Sciences, College of Medicine and Biological Science, Stefan cel Mare University of Suceava, 720229 Suceava, Romania; 2 Department of rheumatology and nephrology, Nicolae Testemițanu State University of Medicine and Pharmacy, Chișinău, Republic of Moldova.; 3 Timofei Moșneaga Republican Clinical Hospital, Chișinău, Republic of Moldova.; 4 Department of Internal Medicine, Scripps Mercy Hospital, San Diego, CA 92103, USA.

**Keywords:** spondyloarthritis, dysbiosis, HLA-B27, autoimmune disease, gut-joint axis, IL-23/17 axis, microorganisms

## Abstract

Ankylosing spondylitis (AS) is a chronic inflammatory disease with complex pathogenesis influenced by genetic, immunological and environmental factors. Recent evidence suggests that gut microbiota significantly contributes to AS etiopathogenesis. Dysbiosis and altered immune responses in the gut potentially trigger or exacerbate the disease through intestinal barrier disruption, alteration of the IL-23/17 axis and metabolite production. This review explores the growing role of gut microbiota in AS and its potential to reshape targeted treatment strategies and facilitate development of adjunct therapies to address disease onset and progression. AS is a multifactorial disease in which gut dysbiosis plays a significant role influencing immune regulation notably through the IL-23/17 pathway. Alterations in gut microbiota composition and its metabolites contribute to systemic inflammation, reinforcing a self-perpetuating feedback loop between gut and spinal inflammation that drives disease progression. Emerging evidence has linked microbial mechanisms to HLA-B27 misfolding promoting endoplasmic reticulum stress and triggering molecular mimicry through gut microbial-associated molecular patterns further contributing to AS pathogenesis. Given the crucial role of gut microbiota in AS, targeting microbiota imbalances presents a promising avenue for novel therapeutic strategies. Although it remains unclear whether gut inflammation and microbial changes precedes AS onset, current evidence suggests an ongoing cycle of autoimmune inflammation involving both the gut and joints. Further research, particularly longitudinal studies, are needed to better understand the gut-joint axis and its potential therapeutic implications in AS management.

## Abbreviations

AIEC - adherent-invasive E. coli,

AS - ankylosing spondylitis,

ASDAS - Ankylosing Spondylitis Disease Activity Score,

axSpA - axial SpA,

BASDAI - Bath Ankylosing Spondylitis Disease Activity Index,

BASFI - Bath Ankylosing Spondylitis Functional Index,

CD - Crohn's disease,

csDMARDs - conventional synthetic disease-modifying antirheumatic drugs,

DCs - dendritic cells,

DPP - dipeptidase protein,

ER - endoplasmic reticulum,

ERAP - ER aminopeptidase,

HCs - healthy controls,

IBD - inflammatory bowel disease,

ILCs - innate lymphoid cells,

LPS - lipopolysaccharide,

MHC - Major Histocompatibility Complex,

NSAIDs - non-steroidal anti-inflammatory drugs,

SCFA - short chain fatty acid,

SpA - spondyloarthritis,

TJ - tight junction,

TNFi - tumor necrosis factor α inhibitors,

UC - ulcerative colitis,

UPR - unfolded protein response.

## INTRODUCTION

Ankylosing spondylitis (AS) is a chronic, immune-mediated inflammatory disorder that is part of a group of conditions, termed spondyloarthritis (SpA), which primarily affect the axial skeleton 1. AS is characterized by inflammation of the sacroiliac joints, spine and their adjacent soft tissues, including tendons and ligaments 2. This disease is clinically characterized by inflammatory back pain, enthesitis (inflammation at tendon and ligament attachment sites), and progressive structural damage 3. Over time, the inflammation can lead to fibrosis, calcification, and ankylosis, particularly in the spine, causing significantly reduced mobility, a characteristic "bamboo spine" radiological appearance in advanced stages and decreased quality of life 4. Recent population estimates suggest that AS affects approximately 0.2-0.5% of the population in the United States. Globally, the age- and sex-adjusted incidence of AS ranges from 0.4 to 14 cases per 100,000 person-years 5. The condition is more prevalent in men than women, with a ratio of 3:1 6. The progression tends to be more severe in men, often characterized by axial involvement, while in women, peripheral joint involvement is more typical and generally milder 7. The onset of AS typically peaks in the second and third decades of life, with approximately 80% of patients experiencing their first symptoms before the age of 30. Diagnosis after the age of 45 is uncommon, occurring in fewer than 5% of cases 8,9. The mean prevalence of AS per 10,000 people, based on data from 36 studies, varies geographically and is estimated at 23.8 in Europe, 16.7 in Asia, 31.9 in North America, 10.2 in Latin America, and 7.4 in Africa 10.

As a chronic condition, AS requires continuous monitoring of patients over time to assess disease progression and treatment response 11. Several standardized scoring systems have been developed to evaluate disease activity and functional status and mobility. The Assessment of Spondyloarthritis International Society (ASAS) criteria are widely used for clinical evaluation. To measure disease activity, tools like the Bath Ankylosing Spondylitis Disease Activity Index (BASDAI) or Ankylosing Spondylitis Disease Activity Score (ASDAS) are commonly applied. Functional capacity and mobility over time are assessed using indices such as the Bath Ankylosing Spondylitis Functional Index (BASFI) or the Bath Ankylosing Spondylitis Measure Index (BASMI). These tools enable accurate tracking of disease dynamics and facilitate personalized management plans to optimize patient outcomes 12. Regular use of these measures allows for timely adjustments to treatment based on diseases activity and functional status. Managing AS involves controlling symptoms, preserving mobility, and preventing damage through pharmacological, and non-pharmacological therapies. Since inflammation is a primary driver of this disease, non-steroidal anti-inflammatory drugs (NSAIDs) are the first-line treatment for reducing inflammation and pain. For peripheral arthritis, conventional synthetic disease-modifying antirheumatic drugs (csDMARDs) are recommended while biologic agents such as tumor necrosis factor α inhibitors (TNFi) and IL-17 inhibitors are reserved for cases where NSAIDs or csDMARDs prove insufficient 7,12.

In addition to chronic inflammation of the spine, sacroiliac joints, entheses, and peripheral joints, AS is frequently associated with extra-articular manifestations, most notably anterior uveitis and microscopic intestinal inflammation 13. AS shares several clinical, pathogenetic, and immunological features with inflammatory bowel diseases (IBD), such as Crohn's disease (CD) and ulcerative colitis (UC), and has a notable genetic predisposition, particularly involving the human leukocyte antigen (HLA)-B27 allele 14,15. Other non-HLA, genetic loci, shared between AS and IBD, have also been identified including interleukin-23 receptor (IL23R) and genes involved in the interleukin-17 (IL17)/interleukin-23 (IL23) pathway 16. Both diseases are characterized by dysregulation of the innate and adaptive immune system involving overproduction of proinflammatory cytokines and T helper cell abnormalities 17. Studies show that nearly 50% of AS patients exhibit subclinical intestinal inflammation, and approximately 5-10% eventually develop clinical IBD 18,19,20,21.

Given the established link between AS and intestinal inflammation, numerous studies have sought to uncover the mechanisms that connect the pathogenesis and progression of these two conditions. Gut microbiota is an important factor in maintaining immune homeostasis with emerging evidence suggesting a link between gut microbiota dysbiosis and AS 22,23. For example, gut microbial dysbiosis such as the decrease of anti-inflammatory bacterial genera like *Faecalibacterium* 24 and accumulation of pro-inflammatory bacteria such as *Klebsiella pneumoniae*, may be associated with AS pathogenesis 25. Further, gut microbiota dysbiosis can trigger alterations in gut barrier integrity, augment immune activation, and promote metabolite imbalance that contribute to systemic inflammation 26. Additionally, microbiota have been implicated in molecular mimicry, with certain bacterial antigens cross-reacting with host antigens to induce autoimmune inflammation 27. As such, there is growing interest in the potential role of adjunct therapies such as probiotics, prebiotics, and dietary modifications aimed at restoring microbial balance and reducing systemic inflammation in autoimmune diseases including SpA. However, specific patterns of gut microbial dysbiosis directly correlated with AS have yet to be fully scientifically validated. Consequently, treatments involving probiotics or other microbiome-targeted interventions remain in the exploratory phase and are not currently recognized as established therapeutic options for AS. Therefore, by synthesizing and reviewing the current evidence regarding the role and mechanisms by which gut microbial dysbiosis influences the development and progression of AS, this review aims to inspire more comprehensive and robust studies to identify the exact microbiota alterations associated with AS. Such research could pave the way for incorporating targeted microbiome therapies into clinical treatment protocols. While these approaches are still theoretical, they hold significant promise in complementing existing management strategies for AS and improve patient outcomes.

## ETIOPATHOGENESIS OF AS 

The etiology and pathogenesis of AS is not completely understood but current evidence suggests that the disease results from a complex interplay of genetic, microbial, environmental, and immunological factors. Genetic predisposition, particularly the strong association with the HLA-B27 gene, is a significant contributor to disease susceptibility, although non-HLA genetic loci have been described as playing a significant role in disease mechanisms 28. Environmental triggers, including infections and alterations in the gut microbiome, are also recognized as important factors in disease onset and progression. These triggers interact with genetic susceptibility to perpetuate immune dysregulation, driving systemic inflammation and, autoimmune responses impacting the axial skeleton, entheses, and peripheral joints, leading to characteristic structural damage seen in AS 13. Additionally, disturbances in bone metabolism, such as excessive bone formation and erosion, further drive disease progression 29,30. The convergence of these factors highlights the interconnected pathways underlying AS, emphasizing the importance of continued research into its pathogenesis to better understand and manage the disease and will be explored in the following subsections.

### Genetic theories, factors and loci in AS

#### HLA-B27 gene role in AS

The role of genetic factors in AS has been recognized since 1961, leading to the discovery of the HLA-B27 gene in 1973. HLA-B27 belongs to the Major Histocompatibility Complex (MHC)-I surface protein encoded by the MHC B gene on chromosome 6 13. Studies have shown that 90-95% of individuals with AS carry the HLA-B27 gene. While the general risk of developing AS among HLA-B27 carriers is approximately 1-2%, this risk increases to 15-20% for those with a first-degree relative diagnosed with AS. The relative risk reaches 94% for first-degree relatives, 25% for second-degree relatives and 4% for third-degree relatives 13,29.

The HLA-B27 gene is highly polymorphic, with approximately 223 subtypes identified as of 2020 30. Its prevalence varies across different ethnic groups, with, for example, Inuit and native Alaskan populations exhibiting the highest rates of HLA-B27 and the highest global prevalence of AS 29,30. The influence of this single gene is one of the most significant genetic associations identified for any complex genetic disease, vastly outweighing the contributions of other known risk genes. The development of the transgenic HLA-B27 rat model provided compelling experimental evidence of HLA-B27's role in AS pathogenesis. These transgenic rats, which co-express human β2-microglobulin (β2M) as a binding partner for MHC class I, spontaneously develop inflammatory conditions that mirror many features of human SpA. This includes a tendency for male predominance, IBD, nail dystrophy, and both peripheral and axial arthritis 31. The precise pathogenetic role of HLA-B27 in AS remains unclear. However, several leading theories have been proposed to explain its involvement in disease development. These include the arthritogenic peptide hypothesis, the misfolded protein hypothesis, and the cell-surface HLA-B27 homodimer hypothesis, each offering unique insights into how HLA-B27 may contribute to AS pathogenesis 13,30. In addition, the hypothesis of ERAP (endoplasmic reticulum aminopeptidase) dysfunction and the hypothesis focusing on alterations in the gut microbiome have been considered 10**. **

MHC class I molecules typically display eight to ten amino acid peptides from inside the cell to CD8+ T cells, helping detect intracellular pathogens. The specific peptide displayed is determined by the structure of the MHC binding groove. In HLA-B27, the peptide repertoire differs from non-disease-associated MHC subtypes. This provides the basis for one of the earlier pathogenic theories, namely the arthritogenic peptide theory, which suggests that HLA-B27 may present self-peptides that mimic pathogen-derived peptides, triggering autoimmunity and cross reactivity. Specifically, this theory represents molecular mimicry and cross-reaction mechanisms 32 and suggests that the presentation of either bacterial peptides by HLA-B27 or self-mimicking HLA-B27-binding peptides from certain bacteria could initiate a cell-mediated immune response leading to chronic inflammation and AS 19. Research has also revealed unique features of HLA-B27, such as flexible peptide binding, but no definitive "arthritogenic peptide" has been found. Additionally, removing CD8+ T cells in HLA-B27 transgenic rats did not prevent disease, indicating other roles for HLA-B27 beyond antigen presentation 31.

The second hypothesis, known as the unfolded protein response (UPR), proposes that HLA-B27 has a tendency to fold slowly and is prone to misfolding within the endoplasmic reticulum (ER) 30. The ER, responsible for protein and lipid synthesis, initiates the UPR as an adaptive stress mechanism when its protein-handling capacity is overwhelmed by excessive or misfolded proteins, including HLA-B27 33. This stress response activates UPR-related genes, which in turn drive the production of pro-inflammatory cytokines such as IL-17, IL-23, and interferon-gamma (IFN-γ) 34. The UPR can be triggered by various external factors like hypoxia, nutrient deprivation, low pH, or oxidized lipids, as well as internal factors such as cellular differentiation or metabolic imbalances. Three key ER-resident stress sensors orchestrate this response: inositol-requiring kinase 1, activating transcription factor 6, and protein kinase RNA-like ER kinase. These pathways work to restore cellular balance by reducing protein load, enhancing folding capacity, or, if stress persists, initiating apoptosis 33. Further, the UPR drives inflammation through multiple pathways, suggesting that ER stress could increase susceptibility to arthritis by amplifying inflammatory responses to infections. This generalized effect may not pose a significant drawback, since a mouse model with systemically elevated IL-23, introduced via genetic mini-circles, successfully mimicked key features of SpA. These included enthesitis, arthritis with new bone growth, and inflammation of the aortic root and skin 35. In addition to its pro-inflammatory effects, the UPR includes mechanisms that reduce stress and inflammation, such as ER-associated degradation (a cellular quality control mechanism within the ER that ensures the proper folding and function of protein) and autophagy (an intracellular catabolic process, important for recycling proteins and larger substrates like aggregates, apoptotic cell remnants, or long-lived and excess organelles, which could otherwise harm cells) 31. The hallmark of autophagy is the formation of double-membrane, cup-shaped structures that engulf cytoplasmic material that deliver it to lysosomes for degradation. Beyond maintaining cellular homeostasis, autophagy eliminates intracellular pathogens and contributes to both innate and adaptive immune responses 36.

A third theory aimed at explaining the role of HLA-B27 in the pathogenesis of AS is the homodimer formation hypothesis. Unlike the typical heterodimeric form of HLA-B27, which includes a β2-microglobulin light chain, the homodimeric form is structurally abnormal and interacts inappropriately with immune cells. Specifically, HLA-B27 heavy chains can form homodimers on the cell surface, which act as pro-inflammatory ligands. These homodimers bind to killer cell immunoglobulin-like receptors (KIRs) expressed on natural killer (NK) cells and T cells, including CD4+ T cells. This binding process triggers abnormal immune activation, leading to the release of pro-inflammatory cytokines, that drive disease progression 19,30,37.

#### Other HLA-B, non-HLA genes connected to AS

Other HLA-B genes such as HLA-B730, HLA-B16, HLA-B35, HLA-B31, HLA-B32, HLA-B38, and HLA-B39 have also been implicated in the development of AS. These genes are associated with HLA-B27-negative AS, although the underlying mechanisms remain unclear 29. In addition, several other genes have been identified as contributors to the pathogenesis of AS, including ERAP1 (endoplasmic reticulum aminopeptidase 1), ERAP2 (endoplasmic reticulum aminopeptidase 2), and NPEPPS (puromycin-sensitive aminopeptidase). These aminopeptidases are critical for the peptide trimming and folding processes of HLA-B27 within the ER. Dysregulation of these aminopeptidases underpins the ERAP dysfunction hypothesis, which posits that improper peptide processing contributes to AS pathogenesis 29. Of these, ERAP1 has been recognized as the second strongest genetic association with AS after HLA-B27. Its primary function is to trim peptides to the optimal length for presentation by HLA-B27. However, loss-of-function variants in ERAP1 can disrupt this process, leading to abnormal HLA-B27 peptide presentation and intracellular accumulation of free heavy chains. This dysregulation in peptide processing may drive inflammation and autoimmune responses associated with AS 30. Another gene locus linked to AS is IL23R, which encodes the interleukin-23 receptor. A specific single nucleotide polymorphism (SNP) in IL23R has been linked to an increased risk of AS and other immune-mediated conditions, including IBD and psoriasis, disorders that frequently co-occur with AS 38.

In addition, the association between toll-like receptor 4 (TLR4) genetic variants and its expression levels suggests that innate immunity contributes to the pathogenesis of AS. TLR4 is a key protein receptor that is activated by endotoxin production, particularly lipopolysaccharides (LPS), secreted from the outer membrane of Gram-negative bacteria, which are characteristically elevated in dysbiosis, a state often observed in AS 39. This association between TLR4 and AS is further evidenced by elevated LPS levels in AS, which correlate with disease activity. This underscores the involvement of TLR4 in driving innate immune and autoinflammatory mechanisms in the disease's pathogenetic process 40. In addition to TLR4, polymorphisms in fucosyltransferase 3 (FUT3) have been closely linked to AS susceptibility, with the rs28362459-G variant, Lewis- negative status, and Le(a-b-) serotype potentially correlating with several aggravated disease indices. In contrast, FUT2 indirectly influences AS pathogenesis by modulating gut microbiota composition and associated inflammatory pathways. While FUT3 has a more specific role through antigen-mediated mechanisms, FUT2 contributes more broadly by shaping microbiota-dependent immune responses 41. Over time, more genes have been associated with AS that include IL12B, RUNX3, EOMES, TBX21, TYK2, CARD9, IL1R1, IL1R2, IL6R, IL7R, IL27, NKX2, and PTGER4 40.

### Immunological factors linking gut inflammation with AS

**Figure 1:**
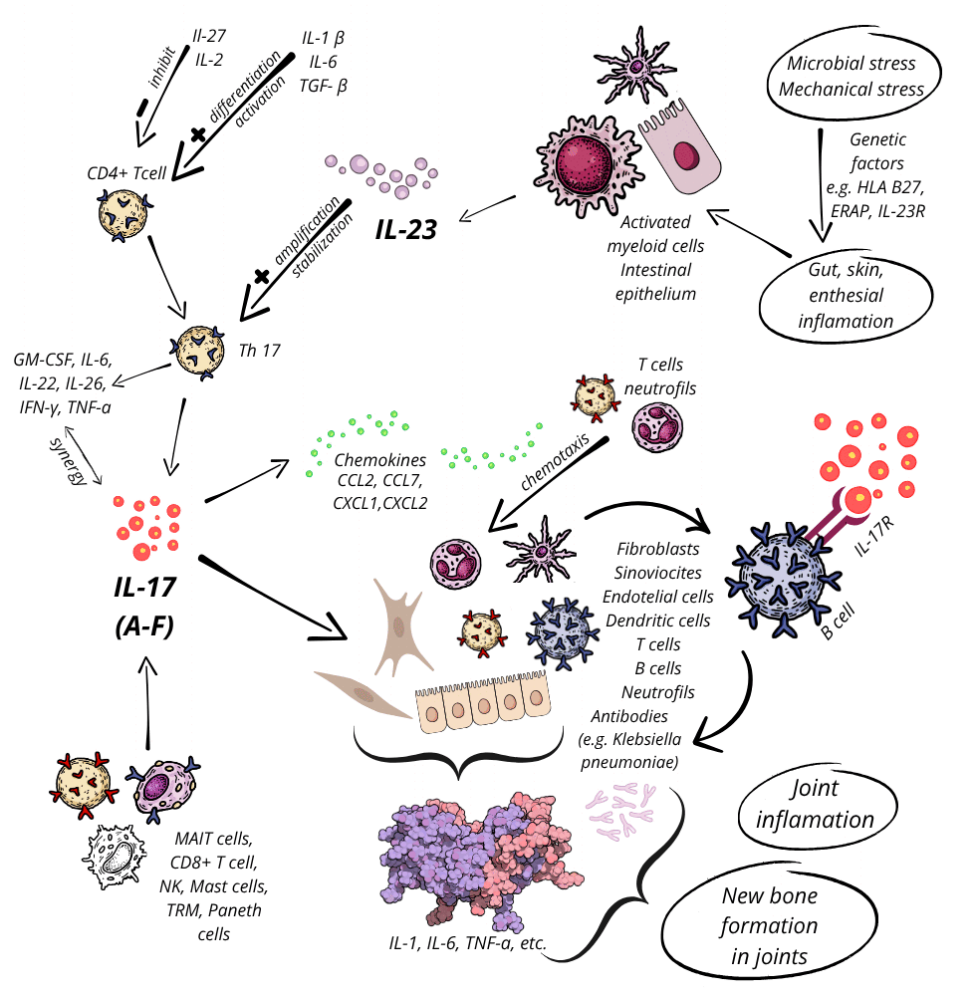
FIGURE 1: The IL-23/IL-17 pathogenetical axis in AS. From left to right: Specific triggers can initiate inflammation in the gut, skin, and entheses of individuals with a genetic predisposition for AS. These triggers activate myeloid cells, such as DCs, macrophages, and intestinal epithelial cells, to secrete IL-23, a heterodimer composed of two subunits, p19 and p40. CD4+ T cells undergo differentiation and activation, influenced by cytokines like IL-1, IL-6, and TGF-β (transforming growth factor-β), to become Th17 cells. IL-23 amplifies and stabilizes Th17 cells, which then produce pro-inflammatory cytokines. Activated Th17 cells secrete IL-17 (variants A through F) and other cytokines, including GM-CSF (granulocyte-macrophage colony-stimulating factor), IL-6, IL-22, IL-26, IFN-γ, and TNF-α, which act synergistically with IL-17 to enhance inflammatory responses. Other cells, such as MAIT (mucosal-associated invariant T) cells, CD8+ T cells, NK cells, mast cells, tissue-TRM (resident memory) cells, and Paneth cells, can also produce IL-17. IL-17 promotes the release of chemokines (e.g., CCL2, CCL7, CXCL1, CXCL2), recruiting inflammatory cells such as neutrophils and T cells to the inflammation site. IL-17 acts on various cell types- fibroblasts, synoviocytes, DCs, T cells, neutrophils, endothelial cells, and B cells- primarily via IL-17R. This binding induces these cells to secrete additional cytokines, perpetuating the inflammatory cycle. Despite AS being classified as a seronegative disease, antibodies (*K. pneumoniae*) secreted by B cells could be involved. Combined with ongoing inflammation, these immune responses contribute to joint inflammation and the characteristic new bone formation in AS (adapted from 32,37,162,163,164,165,166).

Among the majority of inflammatory cytokines involved in the development of AS, the most studied pathway is the IL-17/23 axis (**Figure 1**) 29. This axis has been involved in creating the inflammatory milieu in the gut, which is often observed in AS patients, many of whom report gastrointestinal symptoms and are diagnosed with IBD. IL-23 stimulates the release of pro-inflammatory cytokines and plays a pivotal role in the disruption of the intestinal mucosal barrier, leading to an increased permeability, allowing microbial antigens to penetrate and activate immune responses 42. Further, dendritic cells (DCs) exhibit dysregulated signaling pathways that promote an inflammatory Th17 response. Altered populations of DCs and T cells in AS patients imply a pathogenic role for the IL-23 axis, particularly in fostering intestinal inflammation 43.

Although AS has been known as a "seronegative" condition, indicating the absence of antibodies typically found in autoimmune diseases such as rheumatoid arthritis or systemic lupus erythematosus, emerging evidence points to a role for the adaptive immune system. For example, circulating antibodies have been detected in AS patients with prior *Klebsiella* infections, supporting the molecular mimicry hypothesis and implicating B cell activation in the disease process 44. While innate immunity, especially through the IL-17/23 axis, is thought to initiate AS, the involvement of adaptive immune mechanisms, including B cell-mediated responses, has gained increasing recognition (Fehler! Verweisquelle konnte nicht gefunden werden.). Innate lymphoid cells (ILCs), particularly ILC3, produce IL-17 and IL-22, cytokines known to drive inflammation in AS. One prominent theory posits that IL-17+ and IL-22+ ILC3 migrate from the gut into the systemic circulation, where they are recruited to target tissues, such as the bone marrow, joints, peripheral joints, and entheses. This migration is mediated by integrin-ligand interactions, facilitating the movement of these inflammatory cells to sites of tissue damage, perpetuating chronic inflammation observed in AS 45,46. Elevated serum levels of IL-17 and IL-23 have been observed in individuals with AS compared to healthy controls, with serum IL-17 levels strongly correlating with disease activity and inflammation severity 47.

The autoimmune, innate, and adaptive responses, when dysregulated, affect both axial and gut inflammation, providing a potential link between the pathogenesis of SpA and extra-articular manifestations such as IBD. Therefore, the fifth theory of AS pathogenesis involves the gut microbiota, with dysbiosis and its related immune dysregulation in the gut that may act as trigger for disease onset and progression 48. The subsequent sections will delve into the role of gut microbiota in the context of altered immune response and its impact on the development and progression of AS.

## GUT MICROBIOTA AND AS

### Healthy microbiota and immune-related functions

The human body harbors trillions of microorganisms that coexist in symbiosis with their host, primarily residing in the gastrointestinal tract, skin, saliva, oral mucosa, conjunctiva, and vagina. The intestinal tract alone hosts approximately 10^13^ to 10^14^ microorganisms, including bacteria, archaea, viruses, and fungi. Of these, bacteria constitute more than 99% of the gut microbiota. Under normal conditions, these microbes maintain a dynamic balance through competitive, synergistic, or symbiotic interactions among bacteria, fungi, and viruses 49. The gut microbiota comprises over 1,000 bacterial species classified into six dominant phyla: *Firmicutes*, *Bacteroidetes*, *Actinobacteria*, *Proteobacteria*, F*usobacteria*, and *Verrucomicrobia*. A review 50 mentions over 2172 species and twelve phyla, with the six mentioned phyla accounting for 93.5% of the total microbiome composition. Of these, *Firmicutes *and *Bacteroidetes* are the most abundant, making up approximately 90% of the gut's bacterial population 51 and their ratio (F/B) have attracted significant attention for their critical role in maintaining intestinal homeostasis. An imbalance in F/B ratio, known as dysbiosis, is associated with various disease conditions. For example, an increased F/B ratio is commonly linked to obesity, while a decreased ratio is often observed in IBD 52. In humans, 386 species of bacteria are classified as strictly anaerobic, thriving in low-oxygen environments such as the gastrointestinal mucosa and oral cavity, which support their survival and growth 50.

Proteobacteria*, *a phylum of Gram-negative bacteria containing LPS in their cell membranes, is overrepresented in the gut in several conditions characterized by chronic inflammation, including AS 14. Increased abundance of Proteobacteria is associated with a reduction in *Firmicutes* and overall microbial diversity, a pattern commonly observed in IBD. Bacterial species have been described as anti‐inflammatory (*Bifidobacterium, Lactobacillus, Faecalibacterium prausnitzii*) and pro‐inflammatory (*Bacteroides, Escherichia coli*) 2. Gut homeostasis is also maintained by the dominance of obligate anaerobes belonging to *Firmicutes* and *Bifidobacteriaceae* while an increased presence of facultative anaerobes, particularly from the *Enterobacteriaceae* family, often signals gut dysbiosis 53.

The gut microbiota in a healthy individual varies across different regions of the gastrointestinal tract, with density decreasing from the distal to the proximal colon, and significantly lower levels in the small intestine. The composition is dynamic, evolving over time, determined by factors such as aging and environmental influences like diet, lifestyle, and antibiotic use 51. Moreover, microbiota composition differs significantly between individuals, due to enterotypes, body mass index, and external factors such as exercise frequency, ethnicity, and dietary and cultural habits. A key feature of a resilient microbiome is a compensatory mechanism known as "functional redundancy" 54. This mechanism ensures that similar roles are maintained despite the variations in microbial species. Specifically, functional redundancy allows the microbiota to maintain its overall function and stability even when some species are lost or altered, as other species compensate for their role. This redundancy serves as an important characteristic of a healthy microbiome, contributing to its resilience and ability to adapt to changes, such as dietary shifts or antibiotic treatments 55. In addition to its numerous beneficial roles, the gut microbiota also protects the host against pathogens through the mechanism of competitive exclusion. In this process, nonpathogenic bacteria compete for attachment sites on enterocytes in the gut lining, preventing the adhesion and subsequent invasion of pathogenic entero-invasive bacteria. Furthermore, beneficial bacteria produce antimicrobial substances such as bacteriocins which inhibit the growth of competing pathogens 55.

Of particular relevance, the gut microbiota significantly influences immunomodulatory processes that affect the innate and adaptive immune responses 56. As such, microbiota maintains integrity of the intestinal lining, synthesizes microbial-associated molecular patterns that are recognized by immune cells, activating inflammasomes and promote cytokine production 57. This complex interplay between microbiota and the innate immune system helps regulate gut-associated lymphoid tissue (GALT), ILCs, and phagocytes 58. Microbiota directly and indirectly influence the adaptive immune response via antigen presentation by T-cells and DCs, promotion of T-cell differentiation, B-cell activation, antibody production and the development of immune tolerance 59. Dysregulation of these processes can lead to overactivation of the immune system and cross-reactivity contributing to onset of autoimmune disease such as AS, IBD and reactive arthritis 20,49. Subsequent subsections will explore the specific microbial dysbiosis observed in AS and discuss the mechanisms by which alterations in the microbiota contribute to pathophysiologic changes and disease progression.

### Compositional alterations of microbiota in AS

As previously mentioned, a newer theory regarding the pathogenesis of AS involves alterations in the gut microbiome. However, this area of research is challenging due to the natural variability of microbiota even in healthy individuals, numerous factors affecting its composition. Among these factors, daily habits such as dietary patterns, hydration, alcohol consumption and even sleep can significantly influence the composition of the gut microbiota. Moreover, both short- or long-term use of medication as well as dietary supplementation can disrupt microbial balance. Another critical aspect to consider in microbiome studies in AS is the potential influence of coexisting conditions such as IBD, uveitis, and less well-differentiated forms of spondylitis. These associated diseases can independently alter gut microbiota composition and introduce additional confounding factors.

To overcome this challenge and improve the reliability of microbiome studies in AS, future research should incorporate strategies such as stratification or subgroup analyses, exclusion of patients with significant comorbidities (where appropriate), and thorough adjustment for these variables in statistical models. Additionally, standardized protocols for stool collection (e.g., timing, fasting status) and careful documentation of medications, including antibiotics, NSAIDs, corticosteroids, csDMARDs such as methotrexate and sulfasalazine, and biologic therapies like TNFi (e.g., infliximab, adalimumab) or IL-17 inhibitors (e.g., secukinumab), are essential to reduce variability. Addressing these confounders is crucial to accurately disentangle disease-specific microbiome signatures in AS and to avoid misleading associations that may otherwise arise from the presence of other inflammatory conditions or from the microbiota-modifying effects of these medications.

Nevertheless, important, and generalized trends have been elucidated in the relationship between AS and gut microbial composition. A recent meta-analysis has shown that individuals with AS exhibit decreased microbial richness and evenness as evidenced via the Shannon Index and Simpson Index, respectively 23. Interestingly, those individuals on anti-rheumatic combination treatments demonstrated significant reduction in (-diversity as well, a marker of species diversity within a local area or microbial ecosystem 23. Further, gut dysbiosis is associated with increased disease activity in axial spondyloarthritis (axSpA) 60,61. In a study comparing patients with AS to healthy controls, gut dysbiosis was present in 36% of AS patients versus 17% of controls. This imbalance correlated with higher disease activity scores, including ASDAS and BASDAI, as well as impaired physical function and increased pain levels 60.

Several studies have shown alterations in microbial composition in AS patients. For example, when comparing AS patients with healthy controls (HCs) both groups were dominated by *Firmicutes* and *Bacteroidetes* 62. However, significant differences in their relative abundances have been observed with a significant decrease in *Bacteroidetes* in AS patients compared to HCs, while the levels of *Firmicutes* and *Verrucomicrobia* were notably elevated 63. The dominant bacterial taxa in both AS patients and HCs includes *Bacteroidetes, Firmicutes, Proteobacteria, Fusobacteria*, and *Actinobacteria*. At the phylum level, AS patients have an increased abundance of Proteobacteria and a decreased abundance of *Bacteroidetes* compared to HCs. While increased *Firmicutes* and *Actinobacteria* and decreased *Fusobacteria* were observed in AS patients, it is important to note that these differences were not statistically significant 64. A study involving 97 patients with AS compared to HCs showed elevations in *Actinobacteria* 65. This increased *Actinobacteria* influence the ubiquitination of IκB-α (nuclear factor-kappa B inhibitor alpha), which could activate NF-κB (nuclear factor kappa B) signaling leading to an increase in proinflammatory factors associated with AS 65. Proteobacteria was third in relative abundance, exceeding 15% of all microbiota while *Tenericutes *and *Synergistetes* were decreased in AS 21,62. At the family level, significant differences in the composition of gut microbiota were observed in AS patients compared to HCs, including species belonging to *Lachnospiraceae*, *Veillonellaceae, Prevotellaceae, Porphyromonadaceae,* and *Bacteroidaceae *families 20*. *Similarly, there were increased levels of *Lachnospiraceae, Ruminococcus, Rikenellaceae, Porphyromonadaceae,* and *Bacteroidaceae* in the terminal ileum of AS patients, while *Veillonellaceae *and *Prevotellaceae *were decreased 20*.*

At the genus level, AS patients showed an increased abundance of certain *Firmicutes*, along with a reduced presence of *Prevotella* strain 9 (*Bacteroidetes*),* Megamonas *(*Firmicutes*), and *Fusobacterium *(*Fusobacteria*) 64. Also an increase in the abundance of the *Blautia* genus and a decreased in *Ruminococcus* was observed 62. Furthermore, *Blautia producta*, a *Blautia* species which is increased in AS, was associated with the HLA-B27-positive subgroup. The AS group showed a dominance of *Roseburia,* a finding consistent with the study of Costello *et al*. 7. Among these changes, *Dialister *emerged as the genus with the most significant effect size in AS patients. Similarly, Tito *et al*. reported an increased presence of *Dialister* in the microbiota of inflamed intestinal tissue from biopsies of AS patients 66. A meta-analysis further identified *Dialister* as the most consistently reported genera in axSpA with a positive correlation to ASDAS and a higher prevalence in individuals with radiographic sacroiliitis 67. A study examining the microbiota of AS patients before and after six months of treatment with adalimumab, observed a depletion of *Dialister* in patients prior to treatment. However, after therapy, *Dialister* levels were restored to levels similar to those of HCs, along with restoration to normal abundances of the phyla, *Bacteroidetes* and *Firmicutes* 68. In a different study involving a cohort of AS patients and HCs, eight genera were associated to AS including increased prevalence of *Prevotella* and *Dialister* alongside a reduction in *Bacteroides*. Additionally, a lower proportion of *Ruminococcus gnavus* was observed in individuals with AS 69. Furthermore, a case-control study involving 60 participants (28 with AS and 32 HCs) found a significant reduction in *Clostridium leptum* and an increase in *E. coli *levels in the AS group. No significant differences were observed in other microbial populations between the AS and control groups. However, the AS group exhibited lower microbial diversity, though this difference did not reach statistical significance 2 consistent with other studies 62.

As previously mentioned, *K. pneumoniae*, an opportunistic pathogen found within the human gut, has been suggested to exacerbate the autoimmune disease process. However, the relationship between the fecal microbiome load, particularly *K. pneumoniae*, and AS activity remains controversial. Some researchers propose that *K. pneumoniae* may influence AS development indirectly by interacting with HLA-B27, which will be further discussed in the following sections. Additionally, gut microbiota dysregulations may be linked to a relative deficiency of immune components, resulting in more intense and prolonged immune responses 70.

When analyzing fecal microbiota composition of 150 AS patients, 18 UC patients, and 17 HCs using the GA-map^™^ Dysbiosis Test to assess for similarities and differences 71, there were notable differences in microbial composition across the groups. Compared to HCs, the fecal microbiota of AS patients displayed a higher abundance of Proteobacteria*, Enterobacteriaceae, *Bacilli*, Streptococcus* species, and *Actinobacteria*, coupled with a lower abundance of *Bacteroides *and *Lachnospiraceae*. However, when comparing gut microbiota of IBD and AS, AS patients exhibited a higher abundance of the phylum Proteobacteria, particularly the family *Enterobacteriaceae *and the genera *Shigella* and *Escherichia*, compared to HCs. However, it should be noted that the study found no clear association between the overall fecal microbiota composition and factors such as HLA-B27 status, disease activity, physical function, medication, or smoking 71. Consistent with previous studies, *Enterobacteriaceae*, were enriched in individuals with UC and CD 72*. *Adherent-invasive* E. coli* (AIEC), a strain within *Enterobacteriaceae *that can persist and replicate within epithelial cells and macrophages, is increased in the ileal mucosa of CD patients 73. Similarly, the presence of AIEC and *Prevotella,* has been reported in AS, correlating with gut inflammation and intestinal barrier dysfunction 74.

Li *et al*. 64 explored both bacterial and fungal alterations in the gut microbiota of AS patients, uncovering novel insights into interkingdom interactions. Their study revealed disease-specific changes in the bacterial-fungal network, suggesting that fungi or bacterial-fungal interactions may play a role in AS development. Notably, the study found that fungal dysbiosis was more pronounced than bacterial dysbiosis, with a significant reduction in fungal diversity in AS patients. Furthermore, the abundances of Ascomycota and Basidiomycota were negatively correlated and served as key discriminative features between the AS mycobiota and that of HCs. Although the study lacked sufficient statistical power, these findings highlight a new area of research, emphasizing the need to further investigate interkingdom dynamics 64. Further supporting the role of gut microbiota in AS, studies using germ-free, HLA-B27/β2-microglobulin-transgenic rats have shown that these animals do not develop AS symptoms. However, when commensal intestinal bacteria are introduced into the sterile environment, both intestinal and joint inflammation developed, indicating that gut microbiota plays a role in triggering the disease. In the rat model, there was evidence of impaired mucosal immunity, along with a distinct intestinal dysbiosis 75. Such dysbiosis was characterized by a reduction in *Rikenellaceae *species and an increase in *Prevotella* species 76. Despite these findings, microbiome research in AS remains in its early stages largely due to the need for large patient cohorts and appropriate procedures including sample collection and bacterial DNA sequencing.

**Figure 2:**
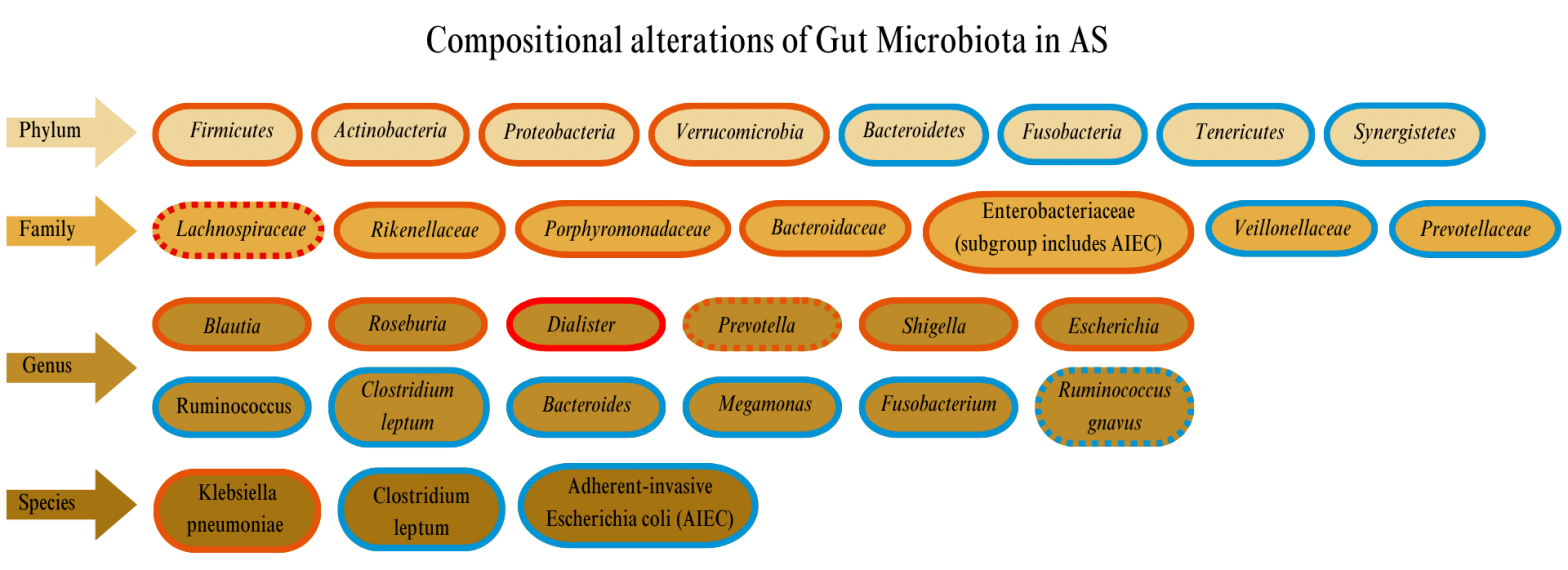
FIGURE 2: Compositional alterations of gut microbiota in AS. Taxonomic distribution (top to bottom). Red border: increased in AS. Blue border: decreased in AS. Red dotted border: generally increased, though some studies report a decrease. Blue dotted border: generally decreased, though some studies report an increase. Brighter red border: most significantly increased in AS.

Additionally, findings by Yang *et al*. challenge the assumption of a direct causal link between AS and gut microbiota. Their study found no genetic-level causal link between the phylum Proteobacteria, the family *Lachnospiraceae*, or the genera *Bacteroides* and *Streptococcus* and AS 77. However, the absence of a genetic association does not necessarily rule out the influence of these microbiota in the development and progression of AS, as gut bacteria likely promote disease pathogenesis through immune regulation within the host. While some studies present contradictory findings, integrating these results remains crucial to developing a comprehensive understanding of the microbial trends within the gastrointestinal tract of AS patients. Changes in the composition profile of gut microbiota in AS are depicted in **Figure 2**.

In microbiome research, particularly in diseases such as AS, the inclusion of large and well-characterized control groups is essential for drawing valid and reproducible conclusions. Using a large control group increases statistical power, reduces the impact of individual outliers, and allows for more robust detection of disease-associated microbial signatures. Equally important is ensuring that the control group is as homogeneous as possible in terms of key confounding variables. Ideally, controls should be matched to patients by age, sex, body mass index, diet, and geographic location, and should not have chronic inflammatory or gastrointestinal diseases, or recent antibiotic or immunosuppressive use. Homogeneity minimizes intra-group variability and enhances the contrast between the disease and control microbiota profiles, making it easier to isolate disease-specific microbial patterns. Without adequately sized and well-matched controls, microbiome studies risk drawing misleading associations that reflect population differences rather than true disease effects.

## MOLECULAR RELATIONSHIPS BETWEEN MICROBIOTA AND HOST IMMUNE SYSTEM IN AS

Understanding the complex interplay between key factors involved in the pathophysiology of AS and the gut microbiota is important for elucidating how they interact to drive disease onset and progression. Mapping these interactions is essential for uncovering potential therapeutic targets and developing strategies aimed at modulating specific pathways for effective disease management. A comprehensive approach should consider the microbiota-HLA-B27 pathway, the interaction between gut microbiota and immune cells, and the role of bacterial metabolites in the disease mechanisms, collectively termed the "microbiota-gut-joint axis". This axis highlights the connection between gut microbiome composition and systemic inflammation, underscoring a link between gut health and the musculoskeletal symptoms characteristic of AS 46. Interestingly, gut dysbiosis may provide additional insights into existing theories of AS pathogenesis and its progression. The following subsections will explore these mechanisms, including molecular mimicry, ER stress, intestinal barrier dysfunction, IL-17/IL-23 pathway, utility of fecal calprotectin and the importance of metabolites such as short chain fatty acids (SCFA).

### Microbiota, molecular mimicry and HLA-B27

Multiple studies support the notion that intestinal microbiota may contribute to the risk of AS through interactions with HLA-B27. As previously hypothesized, human bacterial flora plays an important role in the pathogenesis of HLA-B27-associated diseases, including AS, study evidence reinforce this theory 78. For example, Asquith *et al*. 79 showed that therapies targeting the microbiome may be effective in preventing or treating the diseases associated with HLA genes. They showed that the HLA-B27 genotype was associated with specific changes in the overall gut microbiota composition, favoring a more pro-inflammatory microbiome 79.

The proposed link between gut microbiota and AS pathogenesis through molecular mimicry 30, is based on the observation that pathogenic antigens derived from overgrowth of unfavorable bacterial species within the gut share sequence homology with HLA-B27 80. These bacterial antigens are presented by antigen presenting cells (APC) to the host immune system triggering an immune response 80. Therefore, immune responses generated against pathogenic bacterial species may induce autoimmune-related inflammation against the joints, spine, and surrounding tissues 80. There is significant evidence, both positive and negative, for polypeptide antigens from *K. pneumoniae *and to a lesser extent, other enteric bacteria*, *characteristically elevated in fecal samples of AS patients, in mounting an autoimmune, cross-reactive response against HLA-B27 70,81. Studies from the 1970s and 1980s and reviewed by Zhang *et al*. first elucidated this interesting link 70. Key findings from animal models include anti-HLA-B27 antibodies in rabbits that exhibited significant immune responses against enterobacterial pathogens, such as *Klebsiella* and *Shigella *70. Similarly, follow-up studies demonstrated that allogeneic human sera bind more readily to *K. pneumoniae, Yersinia enterocolitica *and* Shigella sonni, *with anti-*Klebsiella* sera exhibiting lymphotoxic activity against HLA-B27 lymphocytes obtained from patients with AS 82, indicating cross-reactivity between these bacteria and HLA-B27. Interestingly, the cell wall protein, *K43 *on *Klebsiella, *is susceptible to lysis by HLA-B27 positive lymphocytes 83. Further, enzyme immunoassay and radio binding were also used to investigate cross-reactivity, with HLA-B27 positive lymphocytes absorbing significantly more anti-*Klebsiella *antibodies than B27-negative lymphocytes 84. In addition to Gram-negative enteric spp., gram-positive *streptococcal, staphylococcal *and* clostridial spp. *also demonstrated cross-reactivity in HLA-B27 positive AS patients 85. Notwithstanding, study findings by Cameron *et al*. failed to demonstrate cross-reactivity as normal rabbit serum was shown to have a greater binding affinity for peripheral blood mononuclear cells from patients with AS 86. Similarly, Singh *et al*. tested 23 anti-*Klebsiella* sera for cytotoxic activity in HLA-B27 positive AS patients and HLA-B27 negative healthy patients with no specific activity shown against peripheral blood lymphocytes in either group 87.

In recent years, based on the results from human studies, this discussion has been revisited, 88,89. For example, a 2022 meta-analysis study provided support for the presence of increased serum antibodies against *K. pneumoniae *in the feces of AS patients compared to HCs, with notably elevations in IgA and IgG levels, particularly in those with active AS inflammation 88. Similarly, disease activity and functional scores were higher on average in 40 HLA-B27 positive AS patients with elevated fecal *K.* *pneumoniae *levels and serum IgG titers. 89. Additionally, in 2017, Puccetti *et al*. discovered the *K. pneumoniae *dipeptidase protein (DPP), which was detected in 190 out of 200 AS patients, which exhibited sequence homology to fibrocartilaginous sites involved in the AS inflammatory process 90. These findings were not seen in HCs, indicating that approximately 95% of AS patients exhibited serum IgG antibodies against *Klebsiella *DPP. Further supporting this hypothesis, *in vitro* studies have demonstrated that bacterial peptides from microbial species enriched in AS patients stimulate IFN-γ-producing cells, mimicking type II collagen 91. Moreover, studies have shown that *Klebsiella *contain a debranching enzyme pullulanase A (pulA), which has homogenous sequences with collagens I, III and IV as reviewed in detail by Rashid *et al*. 92. Molecular mimicry by pulA has been linked with the development of IBD, particularly CD 93, further providing evidence of a pathogenic link between enteric bacteria, autoimmune intestinal and axial inflammation in AS. Although there has been a resurgence in studies examining this hypothesis in recent years, more data is needed to establish causal relationships between specific bacterial species and autoimmune SpA disease, particularly in humans.

### Microbiota, ER stress and HLA-B27 misfolding

Another mechanistic link between gut microbiota and HLA-B27 involves the ability of unfavorable microbial metabolites to induce ER stress by triggering HLA-B27 misfolding 94. In AS, this misfolding occurs due to formation of aberrant disulfide bonds and protein aggregation 95. Theoretically, these effects may be initiated by bacterial toxins or secreted proteins that directly disrupt ER homeostasis or indirectly contribute to systemic inflammation leading to bacterial overgrowth and increase ER stress in immune and colonic epithelial cells 96. Specifically, HLA-B27 misfolding is driven by activation of UPR or autophagic processes, with cytokine dysregulation and activation of IL-17/IL-23 axis being known activators of the UPR 97.

In the context of autoimmune disease, study have shown that *Salmonella enterica *can activate X-box Binding Protein 1 (XBP1) and AMP-dependent Transcription Factor 6 (ATF6), which are important transcription factors in UPR 98. XBP1 activation facilitates the synthesis of chaperones that assist with protein folding while dysregulation of this pathway has been linked to persistent ER stress and autoimmunity 99. Similarly, ATF6 induces chaperone expression, working synergistically with XBP1 to enhance protein folding, with similar detrimental effects when dysregulated 99. Studies using cell lines showed that *Salmonella *enhanced replication in the presence of HLA-B27 and that the XBP1 pathway is required for efficient *S. enterica *growth 98. This is in line with other reported studies 100,101, which note an increased propensity of HLA-B27 positive patients to exhibit joint inflammatory symptoms following *Salmonella *infection, demonstrating an interesting link between gut microbial dysbiosis and autoimmune inflammation.

Further, autophagic processes on ER stress and UPR have been linked to gut microbial processes in IBD 102, though no direct evidence has linked microbiota to autophagy in AS. Importantly, studies have shown that intestinal biopsies from AS patients exhibited autophagic processes which contributed to HLA misfolding and ER stress 103, despite the absence of UPR gene upregulation. A study by Ciccia *et al*. demonstrated that autophagy was the primary driver of HLA-B27 misfolding and IL-23 expression in inducing subclinical gut inflammation in AS patients, rather than UPR 103. In support of this data, blocking ERAD with proteasome inhibitors in transgenic rodent models, and thereby hampering autophagy, resulted in increased HLA-B27 folding, while activation of autophagy eliminated 50% of misfolded HLA-B27 104. Taken together, these findings provide strong evidence for autophagy as a primary influence in preventing HLA-B27 misfolding. In lieu of this, dysbiosis has been associated with microbiota, specifically increases in pathosymbionts belonging to the *Enterobacteriaceae *family such as *E. coli,* have been associated with dysregulation of autophagic processes in autoimmune diseases 105.

Although there are established links between HLA-B27 misfolding, autophagy and UPR, studies directly supporting the influence of intestinal bacteria in these processes in AS remain limited. A 2025 review has provided additional insights into the role of gut microbiota in modulating UPR and ER stress 96. In summary, exacerbation of intestinal barrier permeability and gut inflammation by pathogenic bacterial species such as *Fusobacterium *and *AIEC *increase UPR activity to help mitigate ER stress. Conversely, beneficial SCFA-producing bacterial species such as *Lactobacillus, Bifidobacterium *and *Lactiplantibacillus *help reinforce barrier integrity via mucin production, limiting XBP1 generation, resulting in less UPR activation 96. Given the mechanistic interactions, it is plausible that reduced XBP1 activation may result in decreased HLA-B27 misfolding. However, the specific role of these microbiota-driven in autoimmune disease pathogeneses has not yet been fully elucidated. The extent to which the human gut microbiota directly contributes to HLA-B27 misfolding, remains unproven and warrants further investigation.

### Microbial dysbiosis, intestinal barrier dysfunction and AS

Gut microbiota dysbiosis leads to increased intestinal permeability, commonly referred to as leaky gut with implications in autoimmune disease 106*. *This occurs due to the disruption of tight junctions (TJ) between epithelial cells, allowing translocation of endotoxins, impairing mucosal immunity, and promoting the release of pro-inflammatory cytokines, ultimately leading to systemic inflammation 106. One key marker of TJ dysregulation is zonulin, a protein that regulates intestinal permeability by modulating TJ integrity 107. Elevated zonulin levels have been observed in the gut epithelium and peripheral blood of AS patients, facilitating the movement of bacteria-derived peptides such as LPS and immune cells into the bloodstream and triggering systemic inflammation 74. This is further evidenced in ileal biopsies of AS patients where adherent and invasive bacteria were noted, accompanying increased serum zonulin and LPS levels 74. Interestingly, LPS-directed IgG and IgA antibodies, that may be driven by *K. pneumoniae, *are elevated 108. Similarly, higher concentrations of LPS-directed IgG antibodies against *E. coli *and *Salmonella typhi *have been detected in AS patients compared to HCs 109, indicating a potential role of bacterial outer membrane protein in AS pathogenesis. Notably, these molecular disruptions can occur even in individuals with histologically normal gut tissue, but are more pronounced in those with chronic inflammation 110.

Conversely, the presence of TJ proteins such as zonula occluden-1 (ZO1), referring to the TJ itself, and occludin, a transmembrane protein that strengthens these TJs, is significantly reduced in AS patients 111. Studies indicate that antagonizing inflammatory bacteria elevates ZO1 and occluding expression, thereby inhibiting TLR-4 activation by LPS and attenuating expression of pro-inflammatory cytokines including TNF-α, IL-17A, IL-6 and IFN-γ 111. These changes were accompanied by reduction of *Enterococcus *and *Escherichia spp. *with concomitant elevation of *Lactobacillus *and *Bifidobacterium *111. These findings were initially described in murine models, where proteoglycan-induced AS mice exhibited increase gut permeability due to lower TJ expression 112. However, biologic therapy for four weeks restored ZO1 and occludin levels, with a corresponding reduction in proinflammatory TNF-α and IL-17, supporting the therapeutic potential of gut barrier modulation in AS management.

**Figure 3:**
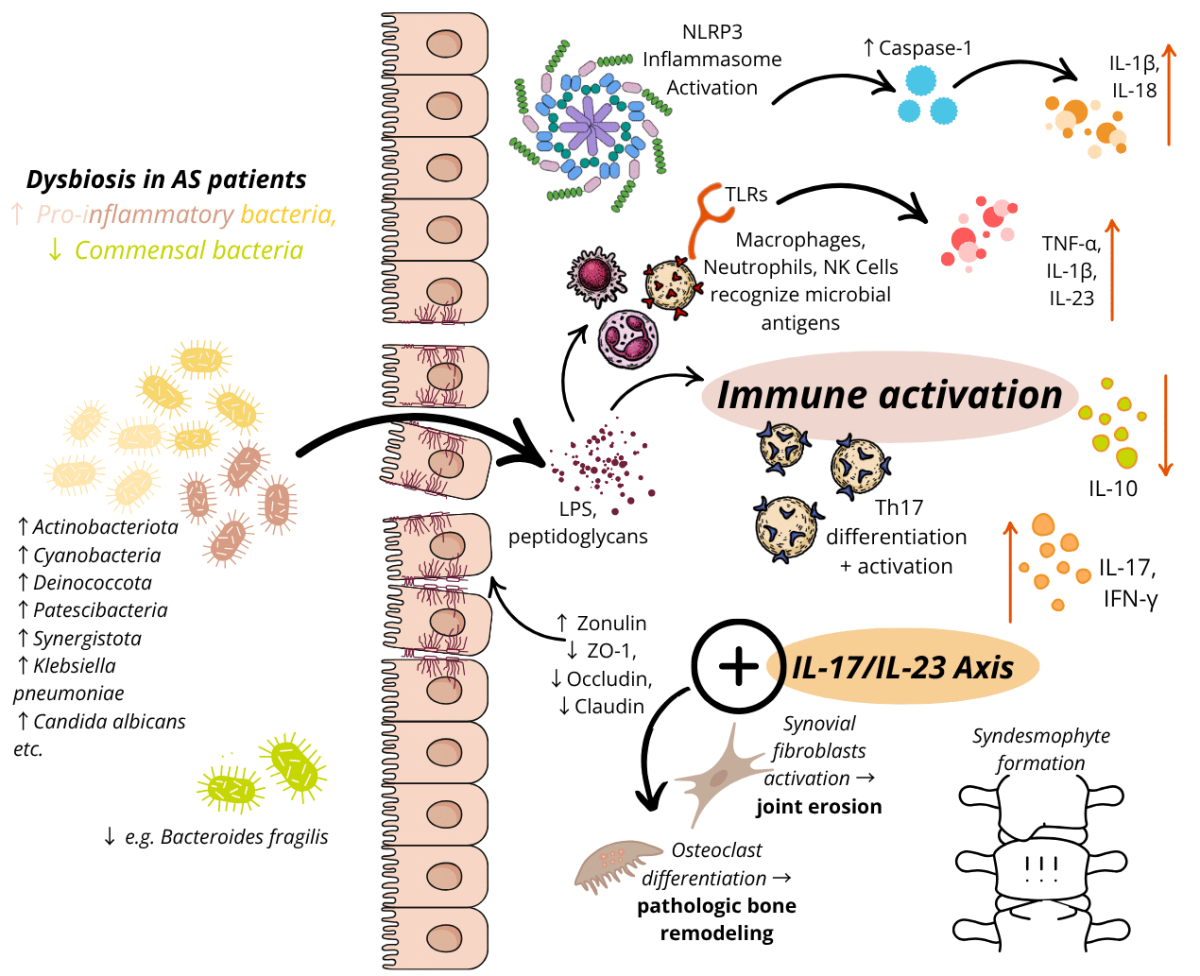
FIGURE 3: Gut dysbiosis and immune activation in AS. Gut microbiota dysbiosis in AS is marked by an increase in pro-inflammatory bacteria* (*shown in orange, yellow, pink) and a decrease in commensal bacteria (blue). Microbial antigens (LPS, peptidoglycans) compromise gut barrier integrity through the dysregulation of zonulin, ZO-1, occludin, and claudin, triggering immune activation via TLRs and the NLRP3 inflammasome. This leads to IL-17/IL-23 axis activation, promoting Th17 differentiation and the recruitment of macrophages, neutrophils, and NK cells that recognize microbial antigens. These processes drive an increase in pro-inflammatory cytokines (TNF-α, IL-1β, IL-17, IL-18, IFN-γ) and a decrease in regulatory cytokines (e.g. IL-10), ultimately contributing to synovial inflammation, osteoclast differentiation (pathologic bone remodeling, syndesmophyte formation), and synovial fibroblast activation (joint erosion).

IL-23 and IL-17 are produced in states of gut inflammation, both in AS and in IBD. IL-17A alone exerts only a modest proinflammatory effect, and is primarily involved in maintaining mucosal immunity and barrier integrity 113. However, when it interacts with other proinflammatory cytokines, it becomes overactivated, and acts as an enhancer of inflammation (**Figure 3**) 37. Biologically, it is imperative to maintain the balance between pathogenic and protective roles of IL-17 signaling. It is believed that tissue damage or the onset of autoimmune diseases occurs when this balance is disrupted, allowing pathogenic IL-17 responses to dominate 114. In this context, gut dysbiosis may activate pathogenic immune cells, perpetuating chronic inflammation, particularly when the intestinal barrier is compromised.

Collectively there is substantial evidence supporting increased intestinal barrier permeability in SpAs including AS 115. However, a key question remains: does intestinal permeability precedes arthritic inflammation, or is it a consequence of joint inflammation? A recent murine study provided insights into this question, suggesting that increased intestinal permeability may precede inflammatory changes within the joints 116. In this study, time course of intestinal inflammation and arthritic changes were assessed at three-time points; preclinical phase (day 4), onset phase (day 11) and acute phase (day 28). In pre-clinical and onset phases, zonulin levels were increased with transient dysbiosis and increased mRNA expression of IL-8, IL-33 and IL-17. The onset phase was characterized by increased TNF-α and IL-23. Notably, while zonulin levels increased at the onset of symptoms, they returned to baseline during the acute phase despite persistence of autoimmune joint inflammation 116. These findings suggest that while gut dysbiosis and increased intestinal permeability may precede arthritic changes, the maintenance of intestinal permeability is not necessarily required for sustaining autoimmune joint inflammation. Overall, this introduces further ambiguity regarding the direct role of the intestinal barrier in the development of SpA, precluding definitive conclusions about causation at this time.

### Microbial dysbiosis, IL-17/IL-23 pathway and AS

Pathogenic insights have demonstrated the importance of the IL-17/IL-23 axis in HLA-B27 SpAs, where IL-23 drives the differentiation of Th17 cells, thereby stimulating IL-17 production 117. Elevated IL-17 levels have been detected in inflamed joints and synovial fluids of AS patients, promoting inflammatory cells recruitment, pro-inflammatory mediators, and bone remodeling 42,117. Additionally, overactivation of this pathway is a downstream effect of several pathogenic mechanisms previously discussed in this review, with Th17 activity and IL-17 production mediating inflammation, which is characteristic of joint stiffness and pain associated with AS. Interestingly, IL-17 functions in a dual capacity depending on its cellular source. IL-23-independent IL-17 production by innate and innate-like lymphocytes appears to play a protective role in maintaining gut homeostasis, whereas IL-23-dependent IL-17 secretion from Th17 cells have been linked to pathogenic effects, contributing to systemic inflammation 110. It has also been documented that Th cells may be primed in the gut and migrate to the joints via shared adhesion molecules found in both IBD and AS, such as (E(7 and (4(7 integrin and chemokine gradients, thereby perpetuating inflammation in axial skeleton 118,119. Notably, colonic biopsies of AS patients showed increased expression of these adhesion molecules in gut mucosal T cell lines, serving as important markers of T-cell trafficking 118.

Given that these processes may originate within the gut, microbiota are intricately related with the IL-17/IL-23 axis with increasing evidence supporting this theory 32. Dysbiosis in AS patients, evidenced by increased levels of phyla *Cyanobacteria*, *Deinococcota*, *Patescibacteria*, *Actinobacteriota* and *Syngergistota* is associated with increased pro-inflammatory cytokines 120. More specifically, a recent study using GWAS data and Mendelian randomization analyses, identified *Actinobacteria *as a mediator of IL-23 and IFN-( response in AS patients 121. Furthermore, *ex vivo* research on human T cells has also shown that *Candida albicans *stimulates Th17 cells to produce IL-17 and IFN-γ, but not IL-10 whereas *Staphylococcus aureus*-activated Th17 cells generate IL-10, which dampens immune responses. These differences likely arise from distinct cytokine environments during the priming phase 46. Functional analysis supports IL-17's role in enhancing epithelial defenses against gram-negative bacteria and fungi. For example, mice lacking IL-17 receptors are significantly more vulnerable to infections by *Klebsiella* and *Candida*. Notably, pathogens such as *Klebsiella, Mycobacterium tuberculosis*, and fungi are potent inducers of Th17 responses 37. For example, *K. pneumoniae *strains, particularly those that are non-virulent have been shown to induce activation of IL-17 and IFN-( genes in pre-activated T helper cells suggesting their role in Th17 differentiation 122.

Another interesting link between the gut microbiota, IL-17/IL-23 pathways and AS is the NLRP3 inflammasome 123. NLRP3 is a key regulator of the innate immune response through activating caspase-1, promoting release of pro-inflammatory cytokines such as IL-1( 124. In AS patients, increased mRNA expression of NLRP3 has been detected in peripheral blood monocytes of patients with AS, along with caspase-1, IL-1(, IL-17 and IL-23 125. Studies using HLA-B27 transgenic rats with AS have shown that dysbiosis directly correlated with NLRP3 inflammasome activity 123. Specifically, NLRP3 expression correlated with induction of IL-23 and IL-17, both of which were IL-1( dependent. Overactivation of NLRP3 was also related to increased disease activity as measured by ASDAS, while NLRP3 blockade prevented iliitis and delayed arthritis onset 123. Therefore, NLRP3 expression is significantly influenced by gut microbiota, is an important driver of autoimmune-mediated inflammation of the joints and axial skeleton, and may be considered as a potential therapeutic target in AS treatment.

### Gut microbiota and fecal calprotectin in AS

Distinct fecal microbiome signatures in AS patients have been linked to gut inflammatory markers such as calprotectin, a protein that reflects neutrophil-driven inflammation in the gut 14. Clinically, fecal calprotectin is a well-established marker used to differentiate IBD from irritable bowel syndrome (IBS) and correlates strongly with clinical, endoscopic, and histological measures of disease activity in IBD. Recent findings also suggest that calprotectin levels may be a marker associated with decreased diseases activity in AS as measured by the ASDAS and BASDAI scores 126. Specifically, following one year of biologic therapy, the initially elevated calprotectin and LPS binding protein levels significantly decrease over the course of treatment.

Further, a longitudinal five-year study 127 explored the role of fecal calprotectin in predicting the IBD development in AS patients. The study found that fecal calprotectin levels were elevated in most AS patients and were associated with disease activity and medication use at both observed time points. Notably, 1.5% of these patients developed CD, with high fecal calprotectin levels emerging as the primary predictor of disease onset. Additionally, studies showed that dysbiosis among AS patients was positively correlated with fecal calprotectin levels (r= 0.303; p< 0.001) with reduction in relative abundances of anti-inflammatory *Faecalibacterium prausnatzii *and *Clostridium *in those with calprotectin > 200 mg/kg 14. AS patients with fecal calprotectin levels >= 200 mg/kg had an increased abundance of the genus *Streptococcus* in their stool samples 14. Interestingly, an increase in *Streptococcus* has also been observed in patients with new-onset CD and has been linked to high recurrence rates of CD. These findings support a significant link between gut inflammation, gut microbiota, and musculoskeletal involvement in AS, underscoring the importance of *Streptococcus* as a potential microbial contributor. The evidence thus far confirms a strong association between calprotectin levels and AS, solidifying its role as a marker of intestinal inflammation. The data further suggests that the gut inflammatory status is connected to the gut microbiota composition in AS patients, highlighting the link between gut dysbiosis and systemic inflammation in AS pathogenesis. Moreover, fecal calprotectin may serve as a valuable biomarker to identify AS patients at risk of developing IBD 127.

In another study, a meaningful correlation was identified between fecal calprotectin levels and various clinical, laboratory, and anthropometric measures, including BASDAI, BASFI, C-reactive protein (CRP), erythrocyte sedimentation rate (ESR), chest expansion, hand-to-ground distance, and the Schober test. Importantly, the cohort of AS patients in this study showed no gastrointestinal symptoms underscoring the fact that IBD and AS are chronic inflammatory diseases of their origin has yet to be fully determined. Despite their distinct clinical presentations, numerous clinical and genetic findings suggest a shared pathogenic connection between the two conditions and that calprotectin is a fast, simple and noninvasive method that can be used instead of the invasive methods like colonoscopy 128. Taken together, the evidence shows that fecal calprotectin levels may act as a biomarker for detecting intestinal dysbiosis in AS patients, even when gastrointestinal symptoms are not present. Regular monitoring of calprotectin levels could help identify specific dysbiosis patterns in AS patients, potentially aiding in early diagnosis and guiding more targeted treatment approaches.

### Gut microbiota, SCFAs and AS

Patients with AS exhibit gut microbiota dysbiosis, characterized by a decreased abundance in SCFA-producing bacteria 129. SCFA are organic acids generated primarily through bacterial fermentation of undigested dietary carbohydrates in the intestinal lumen. The three predominant SCFA, acetate, propionate, and butyrate, constitute more than 95% of total SCFA content typically found in the gut at a ratio of 3:1:1 54,130. While acetate is produced by the majority of gut anaerobes, propionate and butyrate are synthesized by specific bacterial groups through distinct molecular pathways 50. Disruptions in the intestinal microbiota, and the resulting changes in SCFA levels, play a significant role in immunoregulation. These metabolites influence intestinal permeability and the generation of tolerogenic T cells in the gut 131,132. In genetically predisposed individuals alterations in SCFA availability may contribute to the development of AS 133. Moreover, SCFA have been shown to mitigate HLA-B27-associated inflammation as evidenced in animal models, where propionate significantly reduced HLA-B27-driven inflammatory disease 134.

**Figure 4:**
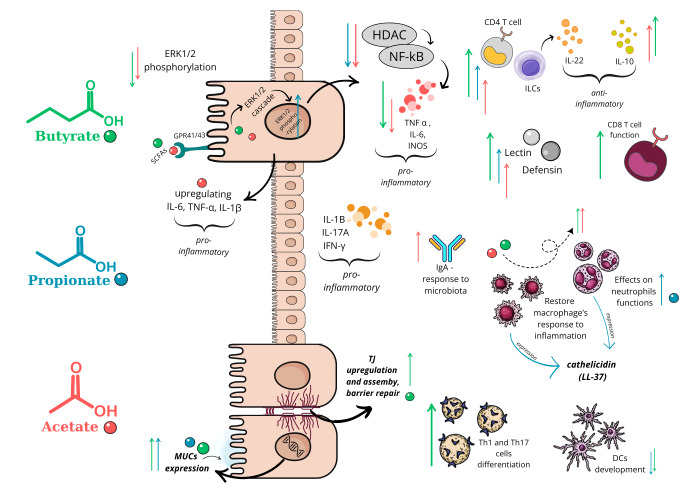
FIGURE 4: Schematic view of SCFA influence on intestinal and immune cells. ↑ enhance/stimulate; ↓inhibit/reduce. All three SCFAs do promote lectin and defensins expression, stimulate CD4+T cells and ILCs to express IL-22, have effects on neutrophils functions. Butyrate (green) upregulates TJ proteins, induces TJ assembly; regulates CD8+T cell’s function; regulates Th1 and Th17 cells differentiation. Butyrate + propionate (blue) block DCs development, enhance MUCs (mucins) expression, promote cathelicidin expression. Propionate reduces proinflammatory cytokines, e.g., IL-1B, IL-17A, and IFN-γ-promoting colonic inflammation. Acetate (red) promotes IgA response to microbiota. Acetate + Butyrate restore macrophage’s response to inflammation; repress ERK1/2 phosphorylation, decrease iNOS (inducible nitric oxide synthase), TNF-α, and IL-6 and enhance IL-10. Acetate + Propionate inhibit HDAC (histone deacetylase) activity and NF-kB activation. Adapted from 54,78,136,167.

In a healthy gut, various bacterial species contribute to SCFA production, with *Firmicutes *phylum playing a key role in butyrate production in the human colon, while *Bacteroidetes* primarily produce acetate and propionate 135,136. Disruptions in the gut microbiota can lead to reduced SCFA production, increasing susceptibility to diseases, as observed in IBD. Fecal sample studies in patients with UC and CD have shown a reduction in butyrate-producing species, accompanied by lower levels of SCFA in the stool 133. Once produced by the colonic microbiota, SCFA serve as a readily accessible energy source for colonocytes. Early research has shown that butyrate oxidation accounts for over 70% of the oxygen consumed by colonocytes in the ascending and descending colon 136. In addition to their role as an energy source for eukaryotic cells, microbial metabolites, SCFA, tryptophan derivatives, vitamin B compounds, and others, play a crucial role in maintaining the integrity of the intestinal environment. They enhance barrier function and exhibit anti-inflammatory properties that support gut health. For instance, virulence factors commonly associated with *Enterobacteriaceae*, such as those produced by *Salmonella *or *Shigella*, promote neutrophil migration across the intestinal epithelium leading SCFA depletion and creating a negative feedback loop that facilitates pathogen proliferation 53. SCFA modulate host immune responses by regulating the expression of pro-inflammatory cytokines. These regulatory effects occur through the activation of macrophages and dendritic cells, influencing both innate and adaptive immunity (**Figure 4**) 53,133.

The mechanisms by which SCFA influence AS pathogenesis involves their role on intestinal barrier integrity, influence on immune responses and even osteoclastogenesis in bones. For example, in 33 SpA patients, *Faecalibacterium prausnitzii, *a known butyrate producer was reduced compared to controls 137. Administration of *F. prausntzii *or butyrate to CD4+ cells derived from SpA patients increased IL-17A differentiation, reduced IL-17 levels and increased IL-10. Further, microbiome-derived butyrate inoculation also reduced osteoclastogenesis in these cell lines and reduced disease activity and inflammation as measured through decreased TNF-α and IL-1B in murine models 137. Osteoclastogenesis, the differentiation of osteoclasts from progenitor cells, is a key driver of pathological bone remodeling in AS, contributing to focal bone proliferation in axial joints, while reducing overall bone mass 138. Propionate and butyrate inhibit osteoclast differentiation through down-regulation of essential osteoclast genes, *TRAF6* and *NFATc1 *139. Therefore, as potent regulators of osteoclast metabolism, SCFA have a protective role in bones of patients with SpA, likely driving the reduced osteoclastogenesis 137.

### Biologics, gut microbiota and AS

The emergence of biologic therapy in treatment of AS, has revolutionized the treatment of AS enabling sustained remission by targeting specific immune pathways 140. TNFi are generally first line biologics with great efficacy in reducing axial inflammation, ethesitis and peripheral arthritis. For patients who fail on TNFi, IL-17i,such as Secukinumab and Ixekizumab serve as alternative immunomodulatory treatments 140. Recent studies highlight the significant influence of biologic therapy on gut microbial composition in AS patients. TNFi therapy has been shown to normalize microbial imbalances, restoring phylum Proteobacteria and family *Pasteurellaceae, *which are elevated in AS patients prior to therapy 141. Importantly, after just one month of TNFi treatment, (-diversity was comparable to that of healthy controls 142. Further, restoration of SCFA-producing bacteria, such as *Megamonsa *and *Lachnoclostridium *was observed in AS patients undergoing TNFi therapy, while corelating with decreased disease severity 129. At the same time, AS promoting genera such as Bacilli and *Haemophilus *were reduced following treatment. Moreover, enrichment of cross-reactive microbial species, as measured through shotgun metagenomics, is reduced to normal levels following TNFi therapy, attenuating potential molecular mimicry from arthritogenic bacterial peptides to improve disease outcomes 143. Studies shown that therapy with Secukinumab treatment induced changes in crucial groups including *Megamonas, Prevotella, Faecalibacterium, Roseburia, Bacteroides *and *Agathobacter, *restoring gut microbial diversity to levels comparable to healthy individuals 144.

Emerging evidence suggests that the efficacy of biologics used in SpA is linked to gut permeability and dysbiosis 145. A study of 48 patients with SpA showed that 21% of those who failed to respond to biologic therapy had higher zonulin levels, frequent antibiotic use and comorbid IBD. Since zonulin expression is a key marker of intestinal permeability, its elevation suggests increased endotoxin leakage leading to persistent immune activation that may impact treatment. Additionally, patients with recurrent antibiotic use have an eightfold increase in biological therapy failure, underscoring the importance of a robust microbial system in treatment success 145, especially when antibiotherapy is associated with other drugs, such as cortisone 146. Further, studies analyzing 61 SpA patients undergoing TNFi therapy showed that non-responders to treatment exhibited higher levels of *Suturella, Clostridia *147 and *Comamonas *68. Although the exact mechanisms by which biologics and gut microbiota exert symbiotic benefits are not fully elucidated, certain hypotheses can be postulated from existing literature. Dysbiosis and inflammation often create a self-perpetuating cycle where inflammation alters microbial composition, and microbiota-driven immune activation exacerbate inflammation. Biologic therapy may contribute to attenuating inflammatory damage on the intestinal barrier, healing of gut lining, thereby reducing gut permeability. However, future studies are needed to explore these mechanisms and whether concomitant microbial support with prebiotics, probiotics and synbiotics in conjunction with biologics may improve treatment efficacy in AS.

## METHODOLOGICAL APPROACHES TO GUT MICROBIOTA PROFILING

Gut microbiota analysis has become a cornerstone of microbiome research, with various methods, each offering distinct advantages and limitations. The most commonly employed techniques include 16S rRNA gene sequencing, shotgun metagenomic sequencing, quantitative PCR (qPCR), and metatranscriptomics, each varying in resolution, complexity, and suitability for specific research applications (**Table 1**).

**Table 1:** Comparative perspective on microbiota sequencing methods.

**Method**	**Strengths**	**Limitations**
16S rRNA sequencing	Cost-effective, high throughput, established pipelines, targeted profiling	Limited taxonomic resolution, primer bias, no functional insights
Shotgun metagenomics	High taxonomic and functional resolution, comprehensive community profiling	High cost, complex bioinformatics, lower throughput
qPCR	High sensitivity, cost-effective for targeted profiling, rapid results	Targeted approach, limited functional insights, potential for bias
Metatranscriptomics	Functional insights, temporal resolution, complementary to metagenomics	High technical complexity, expensive, limited taxonomic resolution

### 16S rRNA sequencing

16S rRNA sequencing is a widely used method due to its relatively low cost and high throughput, making it suitable for large-scale studies 148. This technique targets the hypervariable regions of the 16S rRNA gene, allowing for taxonomic classification of bacteria at the genus or species level 149,150. The bioinformatics pipelines for 16S rRNA data are well-established and widely used, facilitating reproducibility across studies 148.

However, while 16S rRNA sequencing provides good taxonomic resolution at higher levels (e.g., genus), it often fails to distinguish between closely related species due to the conserved nature of the 16S rRNA gene 151,152. Also, primer selection can introduce amplification bias, as some primers may preferentially target specific bacterial taxa, potentially leading to incomplete or skewed representation of microbial community 153. Furthermore, this method does not provide information on the functional potential of the microbiota, as it focuses solely on taxonomic profiling 154.

### Shotgun metagenomic sequencing

Shotgun metagenomics provides both taxonomic and functional insights by sequencing the entire genomic content of microbial communities. It can identify low-abundance taxa and predict metabolic pathways 155. This method captures a broader range of microbial diversity, including bacteria, archaea, viruses, and fungi, making it suitable for studying complex microbial ecosystems 156. Also, shotgun sequencing is more effective at detecting rare taxa compared to 16S rRNA sequencing, especially when sufficient sequencing depth is achieved 152. However, shotgun metagenomics is more expensive and computationally intensive than 16S rRNA sequencing, which can limit its use in large-scale studies 155. Moreover, the analysis of shotgun metagenomic data requires advanced bioinformatics tools, and the accuracy of taxonomic and functional predictions depends heavily on the quality of reference databases 157. In terms of throughput, shotgun metagenomics is less suitable for large-scale studies compared to 16S rRNA sequencing, as it generates significantly larger and more complex datasets per sample 148.

### Quantitative polymerase chain reaction (qPCR)

qPCR is highly sensitive and can detect low-abundance taxa with precision. It is particularly useful for quantifying specific microbial targets 158 and, while it may not provide a comprehensive view of the microbiome, qPCR is cost-effective for targeted profiling of known taxa or functional genes. qPCR is faster than sequencing-based methods, making it suitable for clinical diagnostics and rapid screening applications 159. qPCR requires prior knowledge of the target sequences, limiting its ability to detect novel or unexpected taxa. Like 16S rRNA sequencing, qPCR does not provide information on the functional potential of the microbiota unless combined with other methods 158.

### Metatranscriptomics

Metatranscriptomics identifies actively transcribed genes, providing insights into the functional activity of the microbiota. This makes it particularly useful for studying microbial metabolism and host-microbe interactions 160. It captures the dynamic changes in microbial activity over time, making it suitable for longitudinal studies. When combined with metagenomics, metatranscriptomics provides a more complete understanding of both the composition and function of microbial communities. However, the analysis of metatranscriptomic data is computationally demanding and requires specialized expertise and is more expensive than 16S rRNA sequencing and qPCR due to the need for high-depth sequencing and advanced bioinformatics tools. While it provides functional insights, metatranscriptomics may not offer the same level of taxonomic resolution as shotgun metagenomics 154.

## CONCLUSION AND PERSPECTIVES

AS is a chronic, immune-mediated inflammatory disease primarily affecting the axial skeleton, leading to structural damage and significant impairing in quality of life. The pathogenesis of AS is multifactorial, involving genetic, environmental, and immunological factors. In recent years, gut dysbiosis has emerged as a critical area of investigation, providing novel insights into the disease progression. The genetic predisposition to AS is strongly associated with HLA-B27, with additional contributions from ERAP1, which influences antigen presentation, and IL23R, which modulates immune responses. The IL-23/17 axis plays an important role in linking genetic susceptibility with gut dysbiosis and systemic inflammation, sustaining a cytokine cascade that drives both intestinal and spinal inflammation. Microbial antigens and metabolites, such as LPS, can stimulate IL-23 production in intestinal DCs, which, in turn, activate Th17 cells to secrete pro-inflammatory cytokines. These cytokines not only fuel gut inflammation but also drive spinal inflammation, creating a self-perpetuating cycle that contributes to disease progression.

Gut dysbiosis in AS is further associated with bacterial translocation, a phenomenon exacerbated by increased intestinal permeability ("leaky gut"), which facilitates the systemic spread of inflammatory triggers. Pathogens such as *K. pneumoniae* have been identified as potential triggers for gut inflammation, which then spreads systemically. Elevated fecal calprotectin levels in AS patients further support the presence of gut inflammation correlating with disease activity. From a microbiome perspective, AS patients exhibit a distinct dysbiotic profile, characterized by an increased abundance of *Firmicutes*, *Actinobacteria*, and Proteobacteria while *Bacteroides*, Tenericutes, Synergistetes, and *Ruminococcus gnavus* are reduced. A key finding across studies is the reduction in butyrate-producing bacteria, including *Faecalibacterium*, *Megamonas*, and *Lachnoclostridium*, which are essential for gut barrier integrity and immune regulation. The depletion of these beneficial bacteria may lead to increased intestinal permeability facilitating microbial translocation, and further driving systemic inflammation. Moreover, butyrate has been implicated in osteoclastogenesis and Th17 differentiation, suggesting a direct link between gut dysbiosis and skeletal inflammation in AS.

This review highlights the relation between gut microbiota alterations and AS pathogenesis. The bidirectional relation between gut and spinal inflammation suggests that gut dysbiosis may either precede AS onset or develops concurrently with disease progression. The fact that biologic therapies restore microbial composition supports the hypothesis that systemic inflammation shapes the gut microbiota. Studies using ileocolonoscopy and MRI imaging have demonstrated a strong correlation between chronic gut inflammation and disease severity in axial SpA, even in patients without overt gastrointestinal symptoms. Patients with chronic gut inflammation exhibit higher levels of bone marrow edema, further substantiating the gut-spine connection 161. Additionally, clinical remission of SpA has been associated with normalized digestive histology, whereas persistent arthritis correlates with chronic intestinal inflammation. These findings suggest that gut inflammation is not merely a secondary consequence but may play a primary role in AS pathogenesis. Despite inter-study variability, a consistent dysbiotic signature in AS patients characterized by reduced butyrate-producing bacteria and increased pro-inflammatory species reinforces the role of gut dysbiosis in disease progression. However, the temporal relationship remains unresolved: does gut dysbiosis initiate AS, or does systemic inflammation drive gut microbial alterations? Addressing this question is essential for developing early intervention strategies, in managing AS, potentially altering disease course and improving patient outcomes. By synthesizing current knowledge and identifying key knowledge gaps, this review underscores the need for longitudinal studies exploring gut dysbiosis as a potential early biomarker and therapeutic target in AS. Advancing this research has both scientific and clinical significance, offering hope for improved treatment strategies and better patient outcomes. Understanding the gut-immune-skeletal axis could ultimately redefine AS pathogenesis and treatment, breaking the cycle of chronic inflammation that defines this disease.

## CONFLICT OF INTEREST

The authors declare that there is no conflict of interest concerning the publication of this manuscript.

## References

[1] Sieper J, Poddubnyy D (2017). Axial spondyloarthritis.. Lancet Lond Engl.

[2] Cardoneanu A, Cozma S, Rezus C, Petrariu F, Burlui A, Rezus E (2021). Characteristics of the intestinal microbiome in ankylosing spondylitis.. Exp Ther Med.

[3] Taurog JD, Chhabra A, Colbert RA (2016). Ankylosing Spondylitis and Axial Spondyloarthritis.. N Engl J Med.

[4] Kotsis K, Voulgari PV, Drosos AA, Carvalho AF, Hyphantis T (2014). Health-related quality of life in patients with ankylosing spondylitis: a comprehensive review.. Expert Rev Pharmacoecon Outcomes Res.

[5] Bohn R, Cooney M, Deodhar A, Curtis JR, Golembesky A (2018). Incidence and prevalence of axial spondyloarthritis: methodologic challenges and gaps in the literature.. Clin Exp Rheumatol.

[6] Nelson DA, Kaplan RM, Kurina LM, Weisman MH (2023). Incidence of Ankylosing Spondylitis Among Male and Female United States Army Personnel.. Arthritis Care Res.

[7] Ciurea P (2021). Reumatologie. Vol I.. Editura Medicala, Bucuresti..

[8] Wenker KJ, Quint JM (2024). Reumatologie. vol I.. In: StatPearls.

[9] Johns Hopkins Arthritis
Center (2024). Ankylosing Spondylitis: Symptoms, Diagnosis and Treatment.. https://www.hopkinsarthritis.org/arthritis-info/ankylosing-spondylitis/.

[10] Dean LE, Jones GT, MacDonald AG, Downham C, Sturrock RD, Macfar-lane GJ (2014). Global prevalence of ankylosing spondylitis.. Rheumatology.

[11] Zimba O, Kocyigit BF, Korkosz M (2024). Diagnosis, monitoring, and management of axial spondyloarthritis.. Rheumatol Int.

[12] Ramiro S (2023). ASAS-EULAR recommendations for the management of axial spondyloarthritis: 2022 update.. Ann Rheum Dis.

[13] Zhu W, He X, Cheng K, Zhang L, Chen D, Wang X, Qiu G, Cao X, Weng X (2019). Ankylosing spondylitis: etiology, pathogenesis, and treatments.. Bone Res.

[14] Klingberg E, Magnusson MK, Strid H, Deminger A, Ståhl A, Sundin J, Simrén M, Carlsten H, Öhman L, Forsblad-d’Elia H (2019). A distinct gut microbiota composition in patients with ankylosing spondylitis is associated with increased levels of fecal calprotectin.. Arthritis Res Ther.

[15] Gill T, Asquith M, Rosenbaum JT, Colbert RA (2015). The Intestinal Mi-crobiome in Spondyloarthritis.. Curr Opin Rheumatol.

[16] Zhang Y, Liu W, Lai J, Zeng H (2024). Genetic associations in ankylosing spondylitis: circulating proteins as drug targets and biomarkers.. Front Immunol.

[17] Ding Y, Chen J, Li R, Xue L (2024). Inflammatory bowel disease activity threatens ankylosing spondylitis: implications from Mendelian randomization combined with transcriptome analysis.. Front Immunol.

[18] Benfaremo D, Luchetti MM, Gabrielli A (2019). Biomarkers in Inflamma-tory Bowel Disease-Associated Spondyloarthritis: State of the Art and Unmet Needs.. J Immunol Res.

[19] Garcia-Montoya L, Gul H, Emery P (2018). Recent advances in ankylosing spondylitis: understanding the disease and management.. F1000Research.

[20] Costello M-E, Ciccia F, Willner D, Warrington N, Robinson PC, Gardiner B, Marshall M, Kenna TJ, Triolo G, Brown MA (2015). Brief Report: Intestinal Dysbiosis in Ankylosing Spondylitis.. Arthritis Rheumatol Hoboken NJ.

[21] Chen Z, Qi J, Wei Q, Zheng X, Wu X, Li X, Liao Z, Lin Z, Gu J (2019). Variations in gut microbial profiles in ankylosing spondylitis: disease phenotype-related dysbiosis.. Ann Transl Med.

[22] Chen Z, Qi J, Wei Q, Zheng X, Wu X, Li X, Liao Z, Lin Z, Gu J (2019). Varia-tions in gut microbial profiles in ankylosing spondylitis: disease phenotype-related dysbiosis.. Ann Transl Med.

[23] Su Q-Y, Zhang Y, Qiao D, Song X, Shi Y, Wang Z, Wang C-Y, Zhang S-X (2024). Gut microbiota dysbiosis in ankylosing spondylitis: a systematic review and meta-analysis.. Front Cell Infect Microbiol.

[24] Klingberg E, Magnusson MK, Strid H, Deminger A, Ståhl A, Sundin J, Simrén M, Carlsten H, Öhman L, Forsblad-d’Elia H (2019). A distinct gut microbiota composition in patients with ankylosing spondylitis is associated with increased levels of fecal calprotectin.. Arthritis Res Ther.

[25] Ebringer A (1992). Ankylosing spondylitis is caused by Klebsiella. Evidence from immunogenetic, microbiologic, and serologic studies.. Rheum Dis Clin North Am.

[26] Hou K, Wu Z-X, Chen X-Y, Wang J-Q, Zhang D, Xiao C, Zhu D, Koya JB, Wei L, Li J, Chen Z-S (2022). Microbiota in health and diseases.. Signal Transduct Target Ther.

[27] English J, Patrick S, Stewart LD (2023). The potential role of molecular mimicry by the anaerobic microbiota in the aetiology of autoimmune disease.. Anaerobe.

[28] Zhang Y, Liu W, Lai J, Zeng H (2024). Genetic associations in ankylosing spondylitis: circulating proteins as drug targets and biomarkers.. Front Immunol.

[29] Alexander M (2023). Ankylosing Spondylitis Pathogenesis and Pathophysiolo-gy..

[30] Sharip A, Kunz J (2020). Understanding the Pathogenesis of Spondyloar-thritis.. Biomolecules.

[31] Smith JA (2016). The role of the unfolded protein response in axial spondy-loarthritis.. Clin.

[32] Babaie F, Hasankhani M, Mohammadi H, Safarzadeh E, Rezaiemanesh A, Salimi R, Baradaran B, Babaloo Z (2018). The role of gut microbiota and IL-23/IL-17 pathway in ankylosing spondylitis immunopathogenesis: New insights and updates.. Immunol Lett.

[33] Bettigole SE, Glimcher LH (2015). Endoplasmic Reticulum Stress in Im-munity.. Annu Rev Immunol.

[34] Pedersen SJ, Maksymowych WP (2019). The Pathogenesis of Ankylosing Spondylitis: an Update.. Curr Rheumatol Rep.

[35] Sherlock JP, Joyce-Shaikh B, Turner SP, Chao C-C, Sathe M, Grein J, Gorman DM, Bowman EP, McClanahan TK, Yearley JH, Eberl G, Buckley CD, Kastelein RA, Pierce RH, LaFace DM, Cua DJ (2012). IL-23 induces spondyloarthropathy by acting on ROR-γt+ CD3+CD4−CD8− entheseal resident T cells.. Nat Med.

[36] Lapaquette P, Guzzo J, Bretillon L, Bringer M-A (2015). Cellular and Molecular Connections between Autophagy and Inflammation.. Mediators Inflamm.

[37] Mandour M, Chen S, van de Sande MGH (2021). The Role of the IL-23/IL-17 Axis in Disease Initiation in Spondyloarthritis: Lessons Learned From Animal Models.. Front Immunol.

[38] Nancy Z, Yan L, Hui S, Paul B, Liye C (2021). From the Genetics of Ankylos-ing Spondylitis to New Biology and Drug Target Discovery.. Front Immunol.

[39] Mohr AE, Crawford M, Jasbi P, Fessler S, Sweazea KL (2022). Lipopoly-saccharide and the gut microbiota: considering structural variation.. FEBS Lett.

[40] Mauro D, Thomas R, Guggino G, Lories R, Brown MA, Ciccia F (2021). Ankylosing spondylitis: an autoimmune or autoinflammatory disease?. Nat Rev Rheumatol.

[41] Jiang G, Han R, Chen M, Liu R, Wu M, Zhang X, Ma Y, Yuan Y, Wang R, Shuai Z, Pan F (2020). Associations between fucosyltransferase 3 gene polymor-phisms and ankylosing spondylitis: A case-control study of an east Chinese population.. PLoS ONE.

[42] Paine A, Ritchlin CT (2016). Targeting the interleukin-23/17 axis in axial spondyloarthritis.. Curr Opin Rheumatol.

[43] Rezaiemanesh A, Abdolmaleki M, Abdolmohammadi K, Aghaei H, Pakdel FD, Fatahi Y, Soleimanifar N, Zavvar M, Nicknam MH (2018). Immune cells in-volved in the pathogenesis of ankylosing spondylitis.. Biomed Pharmacother Biomedecine Pharmacother.

[44] Wilbrink R, Spoorenberg A, Verstappen GMPJ, Kroese FGM (2021). B Cell Involvement in the Pathogenesis of Ankylosing Spondylitis.. Int J Mol Sci.

[45] Ciccia F, Guggino G, Rizzo A, Saieva L, Peralta S, Giardina A, Cannizzaro A, Sireci G, De Leo G, Alessandro R, Triolo G (2015). Type 3 innate lymphoid cells producing IL-17 and IL-22 are expanded in the gut, in the peripheral blood, synovial fluid and bone marrow of patients with ankylosing spondylitis.. Ann Rheum Dis.

[46] Schinocca C, Rizzo C, Fasano S, Grasso G, La Barbera L, Ciccia F, Guggino G (2021). Role of the IL-23/IL-17 Pathway in Rheumatic Diseases: An Overview.. Front Immunol.

[47] Serdaroğlu Beyazal M, Tayfun A, Devrimsel G, Yıldırım M, Arpa M (2022). Association of Serum Interleukin-17 and Interleukin-23 Levels with Disease Activi-ty, Function, Mobility, Enthesitis Index in Patients with Ankylosing Spondylitis.. Aktuelle Rheumatologie.

[48] Rosenbaum JT, Davey MP (2011). Time for a gut check: evidence for the hypothesis that HLA-B27 predisposes to ankylosing spondylitis by altering the microbiome.. Arthritis Rheum.

[49] Zhang L, Hu Y, Xu Y, Li P, Ma H, Li X, Li M (2019). The correlation between intestinal dysbiosis and the development of ankylosing spondylitis.. Microb Pathog.

[50] Thursby E, Juge N (2017). Introduction to the human gut microbiota.. Biochem J.

[51] Rinninella E, Raoul P, Cintoni M, Franceschi F, Miggiano GAD, Gasbarrini A, Mele MC (2019). What is the Healthy Gut Microbiota Composition? >A Changing Ecosystem across Age, Environment, Diet, and Diseases.. Microorganisms.

[52] Stojanov S, Berlec A, Štrukelj B (2020). The Influence of Probiotics on the Firmicutes/Bacteroidetes Ratio in the Treatment of Obesity and Inflammatory Bowel disease.. Microorganisms.

[53] Yoo JY, Groer M, Dutra SVO, Sarkar A, McSkimming DI (2020). Gut Microbiota and Immune System Interactions.. Microorganisms.

[54] Rowland I, Gibson G, Heinken A, Scott K, Swann J, Thiele I, Tuohy K (2018). Gut microbiota functions: metabolism of nutrients and other food components.. Eur J Nutr.

[55] Bull MJ, Plummer NT (2014). Part 1: The Human Gut Microbiome in Health and Disease.. Integr Med Clin J.

[56] Belkaid Y, Hand TW (2014). Role of the microbiota in immunity and in-flammation.. Cell.

[57] Yuan C, He Y, Xie K, Feng L, Gao S, Cai L (2023). Review of microbiota gut brain axis and innate immunity in inflammatory and infective diseases.. Front Cell Infect Microbiol.

[58] Jiao Y, Wu L, Huntington ND, Zhang X (2020). Crosstalk Between Gut Microbiota and Innate Immunity and Its Implication in Autoimmune Diseases.. Front Immunol.

[59] Heidari M, Maleki Vareki S, Yaghobi R, Karimi MH (2024). Microbiota activation and regulation of adaptive immunity.. Front Immunol.

[60] Sagard J, Olofsson T, Mogard E, Marsal J, Andréasson K, Geijer M, Kristensen LE, Lindqvist E, Wallman JK (2022). Gut dysbiosis associated with worse disease activity and physical function in axial spondyloarthritis.. Arthritis Res Ther.

[61] Berland M, Meslier V, Berreira Ibraim S, Le Chatelier E, Pons N, Maziers N, Thirion F, Gauthier F, Plaza Oñate F, Furet J-P, Leboime A, Said-Nahal R, Levenez F, Galleron N, Quinquis B, Langella P, Ehrlich SD, Breban M (2023). Both Disease Activity and HLA-B27 Status Are Associated With Gut Microbiome Dysbiosis in Spondyloarthritis Patients.. Arthritis Rheumatol Hoboken NJ.

[62] Oprea M, Cristea D, Dinu S, Ciontea SA, Bojinca VC, Predeteanu D, Balanescu AR, Usein CR (2022). An insight into the fecal microbiota composition in Romanian patients with ankylosing spondylitis using high-throughput 16S rRNA gene amplicon sequencing.. Rev Romana Med Lab.

[63] Liu G, Hao Y, Yang Q, Deng S (2020). The Association of Fecal Microbiota in Ankylosing Spondylitis Cases with C-Reactive Protein and Erythrocyte Sedimen-tation Rate.. Mediators Inflamm.

[64] Li M, Dai B, Tang Y, Lei L, Li N, Liu C, Ge T, Zhang L, Xu Y, Hu Y, Li P, Zhang Y, Yuan J, Li X (2019). Altered Bacterial-Fungal Interkingdom Networks in the Guts of Ankylosing Spondylitis Patients.. mSystems.

[65] Wen C, Zheng Z, Shao T, Liu L, Xie Z, Le Chatelier E, He Z, Zhong W, Fan Y, Zhang L, Li H, Wu C, Hu C, Xu Q, Zhou J, Cai S, Wang D, Huang Y, Breban M, Qin N, Ehrlich SD (2017). Quantitative metagenomics reveals unique gut microbi-ome biomarkers in ankylosing spondylitis.. Genome Biol.

[66] Tito RY, Cypers H, Joossens M, Varkas G, Van Praet L, Glorieus E, Van den Bosch F, De Vos M, Raes J, Elewaut D (2017). Brief Report: Dialister as a Microbial Marker of Disease Activity in Spondyloarthritis.. Arthritis Rheumatol Hoboken NJ.

[67] Wang L, Wang Y, Zhang P, Song C, Pan F, Li G, Peng L, Yang Y, Wei Z, Huang F (2022). Gut microbiota changes in patients with spondyloarthritis: A systematic review.. Semin Arthritis Rheum.

[68] Chen Z, Zheng X, Wu X, Wu J, Li X, Wei Q, Zhang X, Fang L, Jin O, Gu J (2021). Adalimumab Therapy Restores the Gut Microbiota in Patients With Ankylosing Spondylitis.. Front Immunol.

[69] Chislari L (2023). IMPACT OF GUT MICROBIOTA ON ANKYLOSING SPONDYLITIS EVOLUTION.. Arta.

[70] Zhang L, Zhang Y-J, Chen J, Huang X-L, Fang G-S, Yang L-J, Duan Y, Wang J (2018). The association of HLA-B27 and Klebsiella pneumoniae in ankylosing spondylitis: A systematic review.. Microb Pathog.

[71] Klingberg E, Magnusson MK, Strid H, Deminger A, Ståhl A, Sundin J, Simrén M, Carlsten H, Öhman L, Forsblad-d’Elia H (2019). A distinct gut microbiota composition in patients with ankylosing spondylitis is associated with increased levels of fecal calprotectin.. Arthritis Res Ther.

[72] Baldelli V, Scaldaferri F, Putignani L, Del Chierico F (2021). The Role of Enterobacteriaceae in Gut Microbiota Dysbiosis in Inflammatory Bowel Diseases.. Microorganisms.

[73] Zangara MT, Darwish L, Coombes BK (2023). Characterizing the Patho-genic Potential of Crohn’s Disease-Associated Adherent-Invasive Escherichia coli.. EcoSal Plus.

[74] Ciccia F, Guggino G, Rizzo A, Alessandro R, Luchetti MM, Milling S, Saieva L, Cypers H, Stampone T, Di Benedetto P, Gabrielli A, Fasano A, Elewaut D, Triolo G (2017). Dysbiosis and zonulin upregulation alter gut epithelial and vascu-lar barriers in patients with ankylosing spondylitis.. Ann Rheum Dis.

[75] Asquith MJ, Stauffer P, Davin S, Mitchell C, Lin P, Rosenbaum JT (2016). Perturbed Mucosal Immunity and Dysbiosis Accompany Clinical Disease in a Rat Model of Spondyloarthritis.. Arthritis Rheumatol Hoboken NJ.

[76] Lin P, Bach M, Asquith M, Lee AY, Akileswaran L, Stauffer P, Davin S, Pan Y, Cambronne ED, Dorris M, Debelius JW, Lauber CL, Ackermann G, Baeza YV, Gill T, Knight R, Colbert RA, Taurog JD, Van Gelder RN, Rosenbaum JT (2014). HLA-B27 and human β2-microglobulin affect the gut microbiota of transgenic rats.. PloS One.

[77] Yang M, Wan X, Zheng H, Xu K, Xie J, Yu H, Wang J, Xu P (2023). No Evidence of a Genetic Causal Relationship between Ankylosing Spondylitis and Gut Microbiota: A Two-Sample Mendelian Randomization Study.. Nutrients.

[78] Song Z-Y, Yuan D, Zhang S-X (2022). Role of the microbiome and its me-tabolites in ankylosing spondylitis.. Front Immunol.

[79] Asquith M, Sternes PR, Costello M, Karstens L, Diamond S, Martin TM, Li Z, Marshall MS, Spector TD, Le Cao K, Rosenbaum JT, Brown MA (2019). HLA Alleles Associated With Risk of Ankylosing Spondylitis and Rheumatoid Arthritis Influence the Gut Microbiome.. Arthritis Rheumatol.

[80] López-Larrea C, González S, Martinez-Borra J (1998). The role of HLA-B27 polymorphism and molecular mimicry in spondylarthropathy.. Mol Med Today.

[81] Ebringer A (1989). The relationship between Klebsiella infection and ankylos-ing spondylitis.. Baillieres Clin Rheumatol.

[82] Welsh J, Avakian H, Cowling P, Ebringer A, Wooley P, Panayi G, Ebringer R (1980). Ankylosing spondylitis, HLA-B27 and Klebsiella. I. Cross-reactivity stud-ies with rabbit antisera.. Br J Exp Pathol.

[83] Druery C, Bashir H, Geczy AF, Alexander K, Edmonds J (1980). Search for Klebsiella cell wall components cross-reactive with lymphocytes of B27+ AS+ individuals.. Hum Immunol.

[84] Baines M, Ebringer A, Avakian H, Samuel D, James DC (1990). The use of enzyme immunoassay (EIA) and radiobinding assay to investigate the cross-reactivity of Klebsiella antigens and HLA B27 in ankylosing spondylitis patients and healthy controls.. Scand J Rheumatol.

[85] Prendergast JK, McGuigan LE, Geczy AF, Kwong TS, Edmonds JP (1984). Persistence of HLA-B27 cross-reactive bacteria in bowel flora of patients with ankylosing spondylitis.. Infect Immun.

[86] Cameron FH, Russell PJ, Easter JF, Wakefield D, March L (1987). Failure of Klebsiella pneumoniae antibodies to cross-react with peripheral blood mono-nuclear cells from patients with ankylosing spondylitis.. Arthritis Rheum.

[87] Singh B, Milton JD, Woodrow JC (1986). Ankylosing spondylitis, HLA-B27, and Klebsiella: a study of lymphocyte reactivity of anti-Klebsiella sera.. Ann Rheum Dis.

[88] Long F, Wang T, Li Q, Xiong Y, Zeng Y (2022). Association between Klebsiella pneumoniae and ankylosing spondylitis: A systematic review and meta-analysis.. Int J Rheum Dis.

[89] El Maghraby HM, Makram WK, Mohamed HAE, Gerges MA (2023). Human leukocyte antigen - B27 and antibodies to Klebsiella pneumoniae in Anky-losing Spondylitis: associations and clinical outcome.. Egypt J.

[90] Puccetti A, Dolcino M, Tinazzi E, Moretta F, D’Angelo S, Olivieri I, Lunardi C (2017). Antibodies Directed against a Peptide Epitope of a Klebsiella pneumoni-ae-Derived Protein Are Present in Ankylosing Spondylitis.. PloS One.

[91] Zhou C (2020). Metagenomic profiling of the pro-inflammatory gut microbiota in ankylosing spondylitis.. J Autoimmun.

[92] Rashid T, Ebringer A, Tiwana H, Fielder M (2009). Role of Klebsiella and collagens in Crohn’s disease: a new prospect in the use of low-starch diet.. Eur J Gastroenterol Hepatol.

[93] Ebringer A, Rashid T, Tiwana H, Wilson C (2007). A possible link between Crohn’s disease and ankylosing spondylitis via Klebsiella infections.. Clin Rheumatol.

[94] Gill T, Asquith M, Brooks SR, Rosenbaum JT, Colbert RA (2018). Effects of HLA-B27 on Gut Microbiota in Experimental Spondyloarthritis Implicate an Eco-logical Model of Dysbiosis.. Arthritis Rheumatol Hoboken NJ.

[95] Dangoria NS, DeLay ML, Kingsbury DJ, Mear JP, Uchanska-Ziegler B, Ziegler A, Colbert RA (2002). HLA-B27 misfolding is associated with aberrant intermo-lecular disulfide bond formation (dimerization) in the endoplasmic reticulum.. J Biol Chem.

[96] Di Mattia M, Sallese M, Lopetuso LR (2025). The interplay between gut microbiota and the unfolded protein response: Implications for intestinal homeo-stasis preservation and dysbiosis-related diseases.. Microb Pathog.

[97] Colbert RA, Tran TM, Layh-Schmitt G (2014). HLA-B27 misfolding and ankylosing spondylitis.. Mol Immunol.

[98] Antoniou AN, Lenart I, Kriston-Vizi J, Iwawaki T, Turmaine M, McHugh K, Ali S, Blake N, Bowness P, Bajaj-Elliott M, Gould K, Nesbeth D, Powis SJ (2018). Salmonella exploits HLA-B27 and host unfolded protein responses to promote intracellular replication.. Ann Rheum Dis.

[99] Park S-M, Kang T-I, So J-S (2021). Roles of XBP1s in Transcriptional Regu-lation of Target Genes.. Biomedicines.

[100] Ekman P, Kirveskari J, Granfors K (2000). Modification of disease out-come in Salmonella-infected patients by HLA-B27.. Arthritis Rheum.

[101] Tuompo R, Hannu T, Mattila L, Siitonen A, Leirisalo-Repo M (2013). Reactive arthritis following Salmonella infection: a population-based study.. Scand J Rheumatol.

[102] Naama M, Telpaz S, Awad A, Ben-Simon S, Harshuk-Shabso S, Modilevsky S, Rubin E, Sawaed J, Zelik L, Zigdon M, Asulin N, Turjeman S, Werbner M, Wongku-na S, Feeney R, Schroeder BO, Nyska A, Nuriel-Ohayon M, Bel S (2023). Autophagy controls mucus secretion from intestinal goblet cells by alleviating ER stress.. Cell Host Microbe.

[103] Ciccia F, Accardo-Palumbo A, Rizzo A, Guggino G, Raimondo S, Giardina A, Cannizzaro A, Colbert RA, Alessandro R, Triolo G (2014). Evidence that autophagy, but not the unfolded protein response, regulates the expression of IL-23 in the gut of patients with ankylosing spondylitis and subclinical gut inflamma-tion.. Ann Rheum Dis.

[104] Navid F, Layh-Schmitt G, Sikora KA, Cougnoux A, Colbert RA (2018). The Role of Autophagy in the Degradation of Misfolded HLA-B27 Heavy Chains.. Ar-thritis Rheumatol Hoboken NJ.

[105] Larabi A, Barnich N, Nguyen HTT (2020). New insights into the interplay between autophagy, gut microbiota and inflammatory responses in IBD.. Autophagy.

[106] Kinashi Y, Hase K (2021). Partners in Leaky Gut Syndrome: Intestinal Dysbiosis and Autoimmunity.. Front Immunol.

[107] Stevens BR, Goel R, Seungbum K, Richards EM, Holbert RC, Pepine CJ, Raizada MK (2018). Increased human intestinal barrier permeability plasma biomarkers zonulin and FABP2 correlated with plasma LPS and altered gut micro-biome in anxiety or depression.. Gut.

[108] Ahmadi K, Wilson C, Tiwana H, Binder A, Ebringer A (1998). Antibodies to Klebsiella pneumoniae lipopolysaccharide in patients with ankylosing spondyli-tis.. Br J Rheumatol.

[109] Madhavan R, Porkodi R, Rajendran CP, Chandrasekaran AN, Umadevi KR, Alamelu R (2002). IgM, IgG, and IgA response to enterobacteria in patients with ankylosing spondylitis in southern India.. Ann N Y Acad Sci.

[110] Gracey E, Vereecke L, McGovern D, Fröhling M, Schett G, Danese S, De Vos M, Van den Bosch F, Elewaut D (2020). Revisiting the gut-joint axis: links between gut inflammation and spondyloarthritis.. Nat Rev Rheumatol.

[111] Ding M-H, Xu P-G, Wang Y, Ren B, Zhang J-L (2023). Resveratrol Atten-uates Ankylosing Spondylitis in Mice by Inhibiting the TLR4/NF-κB/NLRP3 Pathway and Regulating Gut Microbiota.. Immunol Invest.

[112] Liu B, Yang L, Cui Z, Zheng J, Huang J, Zhao Q, Su Z, Wang M, Zhang W, Liu J, Wang T, Li Q, Lu H (2019). Anti-TNF-α therapy alters the gut microbiota in proteoglycan-induced ankylosing spondylitis in mice.. MicrobiologyOpen.

[113] Rizzo A, Guggino G, Ferrante A, Ciccia F (2018). Role of Subclinical Gut Inflammation in the Pathogenesis of Spondyloarthritis.. Front Med.

[114] Amatya N, Garg AV, Gaffen SL (2017). IL-17 Signaling: The Yin and the Yang.. Trends Immunol.

[115] Hecquet S, Totoson P, Martin H, Prati C, Wendling D, Demougeot C, Verhoeven F (2021). Intestinal permeability in spondyloarthritis and rheumatoid arthritis: A systematic review of the literature.. Semin Arthritis Rheum.

[116] Hecquet S, Totoson P, Martin H, Algros M-P, Saas P, Pais-de-Barros J-P, Atchon A, Valot B, Hocquet D, Tournier M, Prati C, Wendling D, Demougeot C, Verhoeven F (2023). Increased gut permeability and intestinal inflammation precede arthritis onset in the adjuvant-induced model of arthritis.. Arthritis Res Ther.

[117] Jethwa H, Bowness P (2016). The interleukin (IL)-23/IL-17 axis in anky-losing spondylitis: new advances and potentials for treatment.. Clin Exp Immunol.

[118] Van Damme N, Elewaut D, Baeten D, Demetter P, Cuvelier C, Verbruggen G, Mielants H, Veys EM, De Vos M, De Keyser F (2001). Gut mucosal T cell lines from ankylosing spondylitis patients are enriched with alphaEbeta7 integrin.. Clin Exp Rheumatol.

[119] Dai B, Hackney JA, Ichikawa R, Nguyen A, Elstrott J, Orozco LD, Sun K-H, Modrusan Z, Gogineni A, Scherl A, Gubatan J, Habtezion A, Deswal M, Somsouk M, Faubion WA, Chai A, Sharafali Z, Hassanali A, Oh YS, Tole S, McBride J, Keir ME, Yi T (2021). Dual targeting of lymphocyte homing and retention through α4β7 and αEβ7 inhibition in inflammatory bowel disease.. Cell Rep Med.

[120] Liu B, Ding Z, Xiong J, Heng X, Wang H, Chu W (2022). Gut Microbiota and Inflammatory Cytokine Changes in Patients with Ankylosing Spondylitis.. BioMed Res Int.

[121] Du X, Li H, Zhao H, Cui S, Sun X, Tan X (2024). Causal relationship be-tween gut microbiota and ankylosing spondylitis and potential mediating role of inflammatory cytokines: A mendelian randomization study.. PloS One.

[122] Nicolò S, Mattiuz G, Antonelli A, Arena F, Di Pilato V, Giani T, Baccani I, Clemente AM, Castronovo G, Tanturli M, Cozzolino F, Rossolini GM, Torcia MG (2022). Hypervirulent Klebsiella pneumoniae Strains Modulate Human Den-dritic Cell Functions and Affect TH1/TH17 Response.. Microorganisms.

[123] Guggino G, Mauro D, Rizzo A, Alessandro R, Raimondo S, Bergot A-S, Rah-man MA, Ellis JJ, Milling S, Lories R, Elewaut D, Brown MA, Thomas R, Ciccia F (2021). Inflammasome Activation in Ankylosing Spondylitis Is Associated With Gut Dysbiosis.. Arthritis Rheumatol Hoboken NJ.

[124] Blevins HM, Xu Y, Biby S, Zhang S (2022). The NLRP3 Inflammasome Pathway: A Review of Mechanisms and Inhibitors for the Treatment of Inflamma-tory Diseases.. Front Aging Neurosci.

[125] Kim S-K, Cho YJ, Choe J-Y (2018). NLRP3 inflammasomes and NLRP3 inflammasome-derived proinflammatory cytokines in peripheral blood mononu-clear cells of patients with ankylosing spondylitis.. Clin Chim Acta Int J Clin Chem.

[126] Rademacher J, Torgutalp M, Hempel CM, Proft F, Haibel H, Protopopov M, Spiller L, Poddubnyy D, Rios Rodriguez V (2024). Biomarkers reflecting dis-turbed gut barrier under treatment with TNF inhibitors in radiographic axial spondyloarthritis.. RMD Open.

[127] Klingberg E, Strid H, Ståhl A, Deminger A, Carlsten H, Öhman L, Forsblad-d’Elia H (2017). A longitudinal study of fecal calprotectin and the devel-opment of inflammatory bowel disease in ankylosing spondylitis.. Arthritis Res Ther.

[128] Duran A, Kobak S, Sen N, Aktakka S, Atabay T, Orman M (2016). Fecal calprotectin is associated with disease activity in patients with ankylosing spon-dylitis.. Bosn J Basic Med Sci.

[129] Dai Q, Xia X, He C, Huang Y, Chen Y, Wu Y, Chen Y, Hou Q, Shu Y, Zhang W, Xu H, Yin G, Xie Q (2022). Association of anti-TNF-α treatment with gut microbiota of patients with ankylosing spondylitis.. Pharmacogenet Genomics.

[130] Sun M, Wu W, Liu Z, Cong Y (2017). Microbiota metabolite short chain fatty acids, GPCR, and inflammatory bowel diseases.. J Gastroenterol.

[131] Silva YP, Bernardi A, Frozza RL (2020). The Role of Short-Chain Fatty Acids From Gut Microbiota in Gut-Brain Communication.. Front Endocrinol.

[132] Palm NW, de Zoete MR, Flavell RA (2015). Immune-microbiota interac-tions in health and disease.. Clin Immunol.

[133] Scalise G, Ciancio A, Mauro D, Ciccia F (2021). Intestinal Microbial Metabolites in Ankylosing Spondylitis.. J Clin Med.

[134] Asquith M, Davin S, Stauffer P, Michell C, Janowitz C, Lin P, Ensign-Lewis J, Kinchen JM, Koop DR, Rosenbaum JT (2017). Intestinal Metabolites Are Profoundly Altered in the Context of HLA-B27 Expression and Functionally Modu-late Disease in a Rat Model of Spondyloarthritis.. Arthritis Rheumatol Hoboken NJ.

[135] Fusco W, Lorenzo MB, Cintoni M, Porcari S, Rinninella E, Kaitsas F, Lener E, Mele MC, Gasbarrini A, Collado MC, Cammarota G, Ianiro G (2023). Short-Chain Fatty-Acid-Producing Bacteria: Key Components of the Human Gut Micro-biota.. Nutrients.

[136] Parada Venegas D, De la Fuente MK, Landskron G, González MJ, Quera R, Dijkstra G, Harmsen HJM, Faber KN, Hermoso MA (2019). Short Chain Fatty Acids (SCFAs)-Mediated Gut Epithelial and Immune Regulation and Its Relevance for Inflammatory Bowel Diseases.. Front Immunol.

[137] Min HK, Na HS, Jhun J, Lee S-Y, Choi SS, Park GE, Lee JS, Um IG, Lee SY, Seo H, Shin T-S, Kim Y-K, Lee JJ, Kwok S-K, Cho M-L, Park S-H (2023). Identification of gut dysbiosis in axial spondyloarthritis patients and improvement of experi-mental ankylosing spondyloarthritis by microbiome-derived butyrate with im-mune-modulating function.. Front Immunol.

[138] Perpétuo IP, Caetano-Lopes J, Vieira-Sousa E, Campanilho-Marques R, Ponte C, Canhão H, Ainola M, Fonseca JE (2017). Ankylosing Spondylitis Patients Have Impaired Osteoclast Gene Expression in Circulating Osteoclast Precursors.. Front Med.

[139] Lucas S, Omata Y, Hofmann J, Böttcher M, Iljazovic A, Sarter K, Albrecht O, Schulz O, Krishnacoumar B, Krönke G, Herrmann M, Mougiakakos D, Strowig T, Schett G, Zaiss MM (2018). Short-chain fatty acids regulate systemic bone mass and protect from pathological bone loss.. Nat Commun.

[140] Alotaibi A, Albarrak D, Alammari Y (2024). The Efficacy and Safety of Biologics in Treating Ankylosing Spondylitis and Their Impact on Quality of Life and Comorbidities: A Literature Review.. Cureus.

[141] Yuan Y-X, Feng S-R, Wu A-Y, Wu W-H, Tian P, Chen A-Z, Ma X-M, Huang L-L, Yu L (2024). Influence of TNF-α Inhibitors on Gut Microbiota and Immune Modulation in Treating Ankylosing Spondylitis: Insights into Therapeutic Mecha-nisms and Clinical Implications.. J Inflamm Res.

[142] Zhang F, Ma C, Zhang B (2020). Dynamic Variations in Gut Microbiota in Ankylosing Spondylitis Patients Treated with Anti-TNF-α for Six Months.. Ann Clin Lab Sci.

[143] Yin J, Sternes PR, Wang M, Song J, Morrison M, Li T, Zhou L, Wu X, He F, Zhu J, Brown MA, Xu H (2020). Shotgun metagenomics reveals an enrichment of potentially cross-reactive bacterial epitopes in ankylosing spondylitis patients, as well as the effects of TNFi therapy upon microbiome composition.. Ann Rheum Dis.

[144] Sun C, Chao Y, Xu H, Yang X, Pei L, Xu G, Wang F, Fan X, Tang L, Xie C, Su Y, Wang X (2024). Combined analysis of metabolomics and 16S rRNA sequenc-ing for ankylosing spondylitis patients before and after secukinumab therapy.. Int J Rheum Dis.

[145] Chmielińska M, Felis-Giemza A, Olesińska M, Paradowska-Gorycka A, Szukiewicz D (2024). The failure of biological treatment in axial spondyloarthritis is linked to the factors related to increased intestinal permeability and dysbiosis: prospective observational cohort study.. Rheumatol Int.

[146] Cocuz M-E, Cocuz IG, Rodina L, Tataranu E, Caliman-Sturdza OA, Filip F (2024). Treatment with Remdesivir of Children with SARS-CoV-2 Infection: Expe-rience from a Clinical Hospital in Romania.. Life.

[147] Vallier M, Segurens B, Larsonneur E, Meyer V, Ferreira S, Caloustian C, Deleuze J-F, Dougados M, Chamaillard M, Miceli-Richard C (2023). Charac-terisation of gut microbiota composition in patients with axial spondyloarthritis and its modulation by TNF inhibitor treatment.. RMD Open.

[148] Allali I, Arnold JW, Roach J, Cadenas MB, Butz N, Hassan HM, Koci M, Ballou A, Mendoza M, Ali R, Azcarate-Peril MA (2017). A comparison of sequencing platforms and bioinformatics pipelines for compositional analysis of the gut microbiome.. BMC Microbiol.

[149] J Jansen G, P Schouten G, Wiersma M (2024). Advancements in analyt-ical methods for studying the human gut microbiome.. J Biol Methods.

[150] Higashi K, Nakayama J (2015). Profiling of Gut Microbiota Community by Renewed Next Generation Sequencer.. J-STAGE.

[151] Peterson D, Bonham KS, Rowland S, Pattanayak CW, RESONANCE Consorti-um, Klepac-Ceraj V (2021). Comparative Analysis of 16S rRNA Gene and Metagenome Sequencing in Pediatric Gut Microbiomes.. Front Microbiol.

[152] Durazzi F, Sala C, Castellani G, Manfreda G, Remondini D, De Cesare A (2021). Comparison between 16S rRNA and shotgun sequencing data for the taxonomic characterization of the gut microbiota.. Sci Rep.

[153] Waechter C, Fehse L, Welzel M, Heider D, Babalija L, Cheko J, Mueller J, Pöling J, Braun T, Pankuweit S, Weihe E, Kinscherf R, Schieffer B, Luesebrink U, Soufi M, Ruppert V (2023). Comparative analysis of full-length 16s ribosomal RNA genome sequencing in human fecal samples using primer sets with different degrees of degeneracy.. Front Genet.

[154] Kwa WT, Sundarajoo S, Toh KY, Lee J (2023). Application of emerging technologies for gut microbiome research.. Singapore Med J.

[155] Eisenhofer R, Nesme J, Santos-Bay L, Koziol A, Sørensen SJ, Alberdi A, Aizpurua O (2024). A comparison of short-read, HiFi long-read, and hybrid strate-gies for genome-resolved metagenomics.. Microbiol Spectr.

[156] Gulyás G, Kakuk B, Dörmő Á, Járay T, Prazsák I, Csabai Z, Henkrich MM, Boldogkői Z, Tombácz D (2024). Cross-comparison of gut metagenomic profiling strategies.. Commun Biol.

[157] Forry SP (2024). Variability and bias in microbiome metagenomic sequencing: an interlaboratory study comparing experimental protocols.. Sci Rep.

[158] Tully BJ, Finkel SE, Corzett CH (2023). Benchmarking A Novel Quantita-tive PCR-based Microbiome Profiling Platform Against Sequencing-based Meth-ods.. bioRxiv.

[159] Zlobovskaya O, Kurnosov A, Sheptulina A, Glazunova E (2024). Method for quantitative assesment of gut microbiota: a comparative analysis of 16S NGS and qPCR.. Bull Russ State Med Univ.

[160] Gehrig JL, Portik DM, Driscoll MD, Jackson E, Chakraborty S, Gratalo D, Ashby M, Valladares R (2022). Finding the right fit: evaluation of short-read and long-read sequencing approaches to maximize the utility of clinical microbi-ome data.. Microb Genomics.

[161] Praet LV, Jans L, Carron P, Jacques P, Glorieus E, Colman R, Cypers H, Mielants H, Vos MD, Cuvelier C, Bosch FV den, Elewaut D (2014). Degree of bone marrow oedema in sacroiliac joints of patients with axial spondyloarthritis is linked to gut inflammation and male sex: results from the GIANT cohort.. Ann Rheum Dis.

[162] Li H, Tsokos GC (2021). IL-23/IL-17 Axis in Inflammatory Rheumatic Diseases.. Clin Rev Allergy Immunol.

[163] Baeten D, Adamopoulos IE (2021). IL-23 Inhibition in Ankylosing Spondy-litis: Where Did It Go Wrong?. Front Immunol.

[164] Chisălău BA, Cringuș L-I, Vreju FA, Parvănescu CD, Firulescu SC, Dinescu ȘC, Ciobanu DA, Tica AA, Sandu RE, Siloși I, Boldeanu MV, Poenariu IS, Ungureanu AM, Boldeanu L, Bărbulescu AL (2020). New insights into IL‑17/IL‑23 signal-ing in ankylosing spondylitis (Review).. Exp Ther Med.

[165] Gaffen SL, Jain R, Garg AV, Cua DJ (2014). The IL-23-IL-17 immune axis: from mechanisms to therapeutic testing.. Nat Rev Immunol.

[166] Tsukazaki H, Kaito T (2020). The Role of the IL-23/IL-17 Pathway in the Pathogenesis of Spondyloarthritis.. Int J Mol Sci.

[167] Wang RX, Lee JS, Campbell EL, Colgan SP (2020). Microbiota-derived butyrate dynamically regulates intestinal homeostasis through regulation of actin-associated protein synaptopodin.. Proc Natl Acad Sci.

